# Transcriptome analysis of pika heart tissue reveals mechanisms underlying the adaptation of a keystone species on the roof of the world

**DOI:** 10.3389/fgene.2022.1020789

**Published:** 2022-11-23

**Authors:** Danping Mu, Xinlai Wu, Anderson Feijó, Wei Wu, Zhixin Wen, Jilong Cheng, Lin Xia, Qisen Yang, Wenjuan Shan, Deyan Ge

**Affiliations:** ^1^ Xinjiang Key Laboratory of Biological Resources and Genetic Engineering, College of Life Science and Technology, Xinjiang University, Urumqi, China; ^2^ Key Laboratory of Zoological Systematics and Evolution, Institute of Zoology, Chinese Academy of Sciences, Beijing, China; ^3^ Key Laboratory of Zoological Systematics and Application, School of Life Science, Institute of Life Science and Green Development, Hebei University, Baoding, Hebei, China; ^4^ CAS Key Laboratory of Mountain Ecological Restoration and Bioresource Utilization & Ecological Restoration and Biodiversity Conservation Key Laboratory of Sichuan Province, Chengdu Institute of Biology, Chinese Academy of Sciences, Chengdu, Sichuan, China

**Keywords:** high-altitude adaptation, transcriptome analysis, heart tissues, pika, gene expressions, cold tolerance, Qinghai-Tibetan Plateau

## Abstract

High-altitude environments impose intense stresses on living organisms and drive striking phenotypic and genetic adaptations, such as hypoxia resistance, cold tolerance, and increases in metabolic capacity and body mass. As one of the most successful and dominant mammals on the Qinghai-Tibetan Plateau (QHTP), the plateau pika (*Ochotona curzoniae*) has adapted to the extreme environments of the highest altitudes of this region and exhibits tolerance to cold and hypoxia, in contrast to closely related species that inhabit the peripheral alpine bush or forests. To explore the potential genetic mechanisms underlying the adaptation of *O. curzoniae* to a high-altitude environment, we sequenced the heart tissue transcriptomes of adult plateau pikas (comparing specimens from sites at two different altitudes) and Gansu pikas (*O*. *cansus*). Differential expression analysis and weighted gene co-expression network analysis (WGCNA) were used to identify differentially expressed genes (DEGs) and their primary functions. Key genes and pathways related to high-altitude adaptation were identified. In addition to the biological processes of signal transduction, energy metabolism and material transport, the identified plateau pika genes were mainly enriched in biological pathways such as the negative regulation of smooth muscle cell proliferation, the apoptosis signalling pathway, the cellular response to DNA damage stimulus, and ossification involved in bone maturation and heart development. Our results showed that the plateau pika has adapted to the extreme environments of the QHTP *via* protection against cardiomyopathy, tissue structure alterations and improvements in the blood circulation system and energy metabolism. These adaptations shed light on how pikas thrive on the roof of the world.

## Introduction

The Qinghai-Tibetan Plateau (QHTP) in China has an average altitude of more than 4,000 m, the average annual temperature is less than 10°C, and the oxygen concentration is only approximately 50% of the average index on Earth, making it one of the harshest places for animals to live. As a result, species inhabiting the QHTP have evolved distinctive morphological, behavioural, and physiological mechanisms to cope with severe selection pressure, including a lack of oxygen, low temperatures, and intense ultraviolet radiation (Qu et al., 2013b; Zhu et al., 2017; Feijo et al., 2020). The QHTP is therefore an area of great interest to many scholars, and these species have become invaluable models for the comparative analysis of local adaptations. Studies have shown that phenotypic and physiological adaptations for living in extreme environments are recorded at the level of gene transcription (Lan et al., 2018). In particular, studies of high-altitude adaptation have revealed numerous rapidly evolving genes that have undergone natural selection or show different expression patterns in high-altitude species compared to low-altitude inhabitants (Marfell et al., 2013). However, how high-altitude environments shape gene expression patterns remains largely unknown (Tang et al., 2017).

To ensure adequate oxygen to meet the basic needs of life, animals have evolved an elaborate physiological system consisting of respiratory organs (lungs), transport vehicles (erythrocytes), transport channels (blood vessels), and a circulatory power system (heart) (Azad et al., 2017). The role of the heart is to promote blood flow, provide sufficient oxygen and various nutrients to organs and tissues, and remove the final products of metabolism (such as carbon dioxide, inorganic salts, urea, and uric acid), allowing cells to maintain normal metabolism and function (Ream et al., 2008; Azad et al., 2017). Increases in heart rate, blood pressure, and other reactions to altitude can result in changes in cardiac structure and function (Ai et al., 2014). Studies have concluded that an increased heart rate and elevated blood pressure can occur in high-altitude environments, and severe altitude hypoxia can cause myocardial interstitial oedema, degeneration, necrosis, and scar formation (Sahota and Panwar, 2013). The subsequent compensatory increase in cardiac output results in a greater ventricular load, ventricular hypertrophy, a slow heart rate, conduction block, and other arrhythmias (Dor et al., 2001; Zhang et al., 2017).

As adaptations to the harsh high-altitude environment, the ability to promote the expression of genes related to adaptation to hypoxic conditions, protect cardiac muscle cells and tissue structure, and increase blood circulation has been observed in the indigenous people of the QHTP (Zhang et al., 2017). Moreover, Guan et al. (2017) found that yak (*Bos grunniens*) has larger lung and heart tissues than cattle (*Bos taurus*), and the differential miRNAs between these two tissues show synergistic effects playing a significant role in high-altitude adaptation. Zhang et al. (2017) found that the *HIF-1* signalling pathway, cardiovascular development, and *VEGF* signalling pathway may play critical roles in hypoxia adaptation. Overall, these studies reveal genes and pathways related to high-altitude adaptation and demonstrate that cardiopulmonary organs show adaptive transcriptional changes in high-altitude environments. Nevertheless, the heart gene expression patterns of animals at high altitudes are not completely clear, and additional systematic and in-depth studies of the adaptive strategies and regulation mechanisms of animal cardiopulmonary organs in this environment are needed.

Pika (*Ochotona* spp.) is a small nonhibernating herbivorous mammal distributed on the QHTP, in its vicinity and in mountains of Central Asia, Northeast Asia, and North America (Gureev, 1964; Su and Liu, 2000; Melo Ferreira et al., 2015; Koju et al., 2017; Solari and Hadly, 2018). They are highly adapted to cold and alpine environments (Wang et al., 2020b). In addition, the plateau pika (*O. curzoniae*) is one of the most dominant keystone mammal species on the QHTP (Wilson and Smith, 2015; Jukes, 2018; Smith et al., 2019). Studies have demonstrated that plateau pikas have evolved several adaptations to low-temperature and low-oxygen conditions (Montuire, 2001; Liu et al., 2012; Wang et al., 2020b). Qi et al. (2008) showed that plateau pikas can improve their adaptation to anoxic environments by increasing the density of heart mitochondria and the microvasculature and myoglobin contents. Zhu et al. (2021) measured the resting metabolic rate and transcriptional expression levels of adipose tissue, liver and skeletal muscle in pikas along different elevation gradients and proved that plateau pikas could adapt to the extreme environment of the QHTP through increases in the expression of thermogenesis genes and energy metabolism. However, current research on the high-altitude adaptation of pikas is mainly focused on nonshivering thermogenesis (Wang et al., 1999; Wang et al., 2006), the regulation of skeletal muscle lactate dehydrogenase-c, liver hypoxia and pulmonary circulation (Liu et al., 2012; Melo Ferreira et al., 2015; Pichon et al., 2015; Zhu et al., 2021). The role and adaptive mechanisms of cardiac tissue in extreme environments are less well studied, and most of the relevant previous studies have been focused on single species of pikas, without intraspecific and interspecific comparisons.

The life history of a species is the product of natural selection and can reflect the continuous adaptation of a species to its habitat. It is important to further explore the adaptive evolution of species by analysing the differences in closely related species in distinct habitats (Qu et al., 2013a). Previous studies have shown that pikas originated on the QHTP and gradually expanded to Eurasia and North America (Wang et al., 2020b). Among extant species, the Gansu pika (*O. cansus*) mainly inhabits the alpine bushes and forest margins of the mountainous coniferous and broad-leaved mixed forest belt from altitudes of 2,200–4,000 m. This species excavates burrows near roots, ridges in grass fields and piles of rocks (Su et al., 2005). However, the plateau pika mainly lives in alpine meadows and open grasslands from altitudes of 3,100–5,100 m, and its distribution on the QHTP is the most extensive among pika species, obviously exceeding that of its congeneric species (Smith and Foggin, 1999). Therefore, in this study, cardiac tissues of Gansu pika and plateau pika, two closely related species, were selected for a comparative transcriptome analysis.

Here, we explore the potential genetic mechanisms underlying adaptation to high-altitude environments in pikas by analysing cardiac transcriptomic differences. Heart tissues of plateau pika and Gansu pika were collected for transcriptome sequencing, and the differentially expressed genes (DEGs) and their main functions were identified through differential expression analysis and weighted gene co-expression network analysis (WGCNA). The results showed that there were considerable differences in the expression patterns between plateau pikas and Gansu pikas. In addition, we identified slight differences between plateau pikas from different altitudes. The genes identified *via* differential expression analysis and WGCNA were mainly enriched in signal transduction, energy metabolism, material transport, negative regulation of smooth muscle cell proliferation and heart development.

## Materials and methods

### Sequencing material collection

**FIGURE 1:**
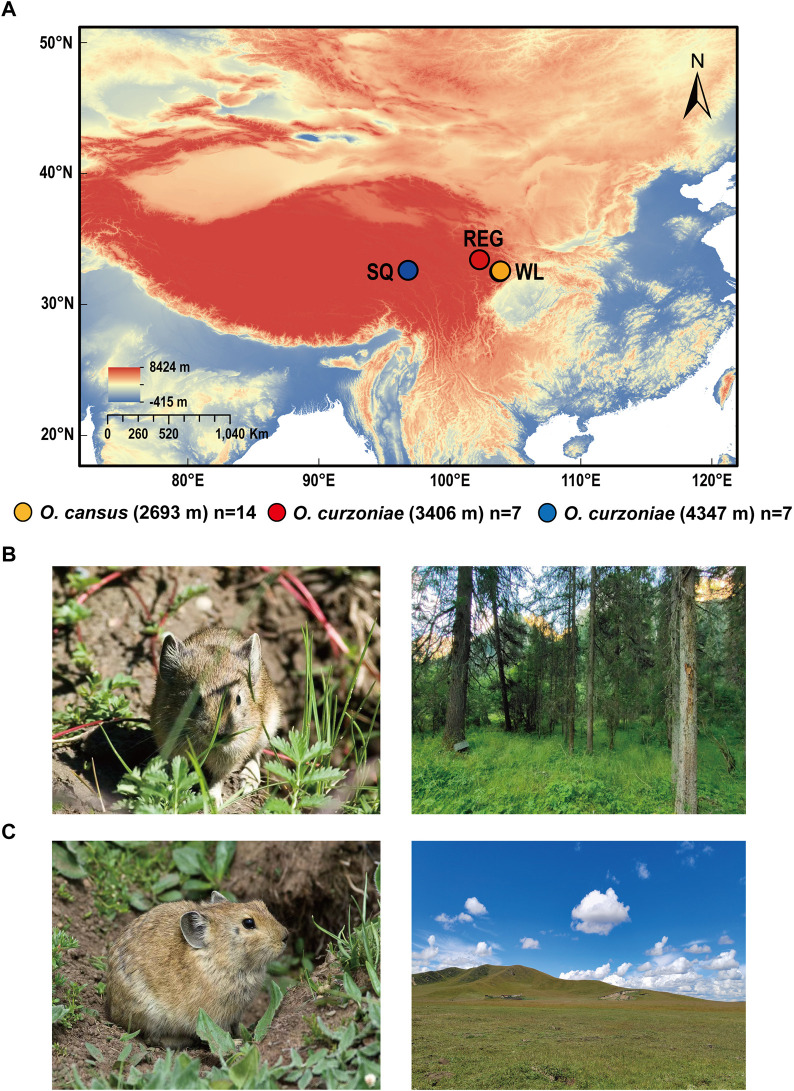
Diagram of pika sample collection sites. **(A)** Collection of pika samples at WL, REG and SQ. **(B)** *O*. *cansus* (From Andrey Lissovsky) and collection site in the Wanglang National Nature Reserve (From Danping Mu). **(C)** *O. curzoniae* (From Andrey Lissovsky) and collection site in the Zoige Wetland National Nature Reserve (From Danping Mu).

Fourteen *O*. *cansus* adults were collected in Wanglang National Nature Reserve (WL, N = 32.9050, E = 104.0540, 2,693 m), seven *O*. *curzoniae* adults were collected in Zoige Wetland National Nature Reserve (REG, N = 33.6499, E = 102.8204, 3,406 m), and seven *O*. *curzoniae* were collected in Shiqu (SQ, N = 32.9950, E = 98.4420, 4,347 m) (Figure 1; Supplementary Table S1). We collected pika heart tissue, cut it into small pieces and preserved the obtained tissue in RNA preservation solution for 4 h at room temperature. The tissue was then frozen in liquid nitrogen for 10 min and stored at −80°C for subsequent RNA extraction. Voucher specimens were preserved in the National Zoological Museum, Institute of Zoology, Chinese Academy of Sciences.

### RNA extraction and transcriptome sequencing

Transcriptome sequencing was conducted on 28 heart tissue samples from two pika species (Supplementary Table S1). Total RNA was extracted from heart tissues using the RNeasy Fibrous Tissue Mini Kit (Tiangen Biotech, Beijing, China) according to the manufacturer’s instructions (Hao et al., 2019). We used 2 μg of RNA from each sample as the input material for sequencing. Before library preparation, we checked RNA integrity, purity, and concentrations. RNA purity was checked with a Nanodrop 1000 UV instrument (Thermo Fisher Technologies, United States). RNA degradation and contamination were monitored by agarose gel electrophoresis. A Qubit™ 4.0 Fluorometer (Invitrogen ABI, Palo Alto, CA, United States) was used to measure the concentration of RNA, and an Agilent 2100 Bioanalyzer (Agilent Technologies, Palo Alto, CA, United States) and associated RNA 6000 Nano kits were used to assess and quantify the quality of RNA. Subsequently, the mRNA was isolated with Oligo (dT) magnetic beads, and the fragments were used to synthesize cDNA. After purification and terminal repair, an A (adenine) nucleotide was added. The mRNA fragments were linked with an adapter and amplified by PCR. Sequencing was conducted on the Illumina NovaSeq HiSeq platform to generate 150 bp paired-end reads. Sequencing was conducted by Berry Genomics (Beijing).

### Transcriptome quality controls and quantitative Salmon analysis

The quality control and preprocessing of the data were carried out by using FastQC V0.11 (Andrews, 2010). Fastp V0.23.1 (Chen et al., 2018) was used to filter adaptors and poor-quality reads. Then, Salmon V1.5.2 (Patro et al., 2017) was used to quickly quantify transcript expression, and the genome assembly of *O*. *curzoniae* (RefSeq GCF_017591425.1, NIBS_Ocur_1.0 from NCBI) was used as a reference. The expression of transcripts was estimated from dual-end sequencing data, and the “quant.sf” quantification file was the output file. The transcripts per million (TPM) method was chosen to represent the expression level of each individual gene.

### Differential expression analysis of RNA-Seq datas

Salmon was utilized to calculate gene expression levels in 28 individuals, and the Salmon results were then normalized and log transformed. We obtained 32,693 genes to construct the dds matrix with the DESeq2 V1.34.0 R package (Love et al., 2014) to analyse differential expression in the heart tissues. In particular, we used the DESeq2 package to conduct differential expression analysis based on the negative binominal distribution. Prior to the differential expression analysis, a PCA was performed on the standardized expression results of 14 samples (five females and nine females) of Gansu pikas to examine the influence of sexual dimorphism.

The *p*-value generated by Benjamin and Hochberg’s calculation method (false discovery rate, FDR) (Storey, 2002; Storey et al., 2004; Bin and Steve, 2005), was used as the parameter for screening DEGs (Benjamini and Hochberg, 1995; Maurano et al., 2012). DEGs were screened according to an FDR < 0.01 and a |fold change (FC)| > 2. DEGs were visualized in a volcano plot by using the R package “ggplot2 (V3.36)” (Yang et al., 2021) and in a heatmap plot by using the R package pheatmap V1.0.12 (Kucukural et al., 2019). PCA was performed after the dds matrix was normalized in the above steps. The first two PCA axes were used to visualize the differentiation among individuals (Bao et al., 2021). The selected DEGs were used for subsequent functional enrichment analysis.

### Weighted gene coexpression network analysis

We calculated gene expression levels from the raw counts of each sample, and TPM values calculated by Salmon were used as a measure of transcript abundance to construct a gene expression matrix for 28 samples. The transformation of the gene expression matrix was normalized using the variance-stabilizing transformation (VST) included in DESeq2 (Bin and Steve, 2005). Herein, the top 20% of upregulated genes were selected for WGCNA (Zhu et al., 2019; Bai et al., 2020; Liu et al., 2020; Gong et al., 2022; Wang et al., 2022). A weighted gene coexpression network was constructed using the WGCNA V.1.66 R package (Langfelder and Horvath, 2008) to ensure that the maximum number of genes related to altitude were obtained. A network based on the approximate scale-free topology was constructed by selecting the most suitable soft threshold, which resulted in a scale-free R^2^ fit. We then performed average linkage hierarchical clustering with TOM-based dissimilarity to construct a dendrogram, setting 0.3 at the height cut-off and 25 as the minimum module size. Modules with a higher correlation were merged (r < 0.25). Each module was assigned different colours for visualization. We calculated the first principal components as a measure of module expression. Genes with similar expression patterns were grouped into a coexpression module with a specific molecular mechanism. Then, the expression levels were accompanied by the trait data, and association analysis between module genes and traits was conducted. Spearman’s correlation was used to analyse the correlation between characteristic module genes and altitude, and the module with the highest correlation was selected as the module related to altitude adaptation (Langfelder and Horvath, 2008). The hub genes in the elevation-related module were extracted based on the criteria of a kME value greater than 0.8 and a significance of genes and traits greater than 0.2 (Wu et al., 2016). Scatter plots were generated to illustrate the maximum module (MM) and gene significance (GS) genetically related to altitude (Pei et al., 2017) based on the cut-off criteria of |MM| > 0.8 and |GS| > 0.2, which were extracted from the centers in Gene Modules of Interest (Tang et al., 2018). Hub genes are those that show high connectivity in the network and play crucial roles in biological processes and influence the regulation of other genes in related pathways (Bao et al., 2021).

### Gene functional enrichment and pathway analysis

To assess the gene functions and metabolic pathways of the screened hub genes, we used ShinyGO V0.75 (Ge et al., 2020) for the functional enrichment analysis of the selected genes obtained from the above analyses. ShinyGO is a Shiny application developed based on several R/Bioconductor packages and a large annotation and pathway database compiled from many sources, with graphical visualization of enrichment, pathways, gene characteristics and protein interactions. The American pika was chosen as a background species with a *p*-value cut-off (FDR) < 0.05 as the major criterion for selecting the significantly enriched biological pathways, and the *p* < 0.05 value corrected using the Benjamini & Hochberg algorithm was set as the threshold for identifying biological processes and pathways. In addition, the intersection dataset of DEGs and hub genes related to altitude was retrieved, and the genes screened *via* the two methods were analysed to determine the mechanism whereby pika heart tissue has adapted to the plateau environment.

## Results

### Results of transcriptome sequencing and Salmon analysis

We obtained a total of 106 Gb of clean read files for the 28 heart samples. The average number of clean reads was 23,415,709.86, and the average amount of data was approximately 7.0 GB. The average GC content of the 28 samples was 50.7%, indicating that the transcriptome sequencing data were of high quality and could be further analysed (Supplementary Figure S1; Supplementary Table S2). The quantitative results were filtered to remove genes with read counts of less than half the total number of samples, and the expression results of 32,693 standardized genes were obtained.

### Gene expression and cluster analysis

PCA on samples of O. cansus from Wanglang revealed insignificant differences between female and male (Supplementary Figure S2). Then, we combined both gender in the following analyses.

**FIGURE 2:**
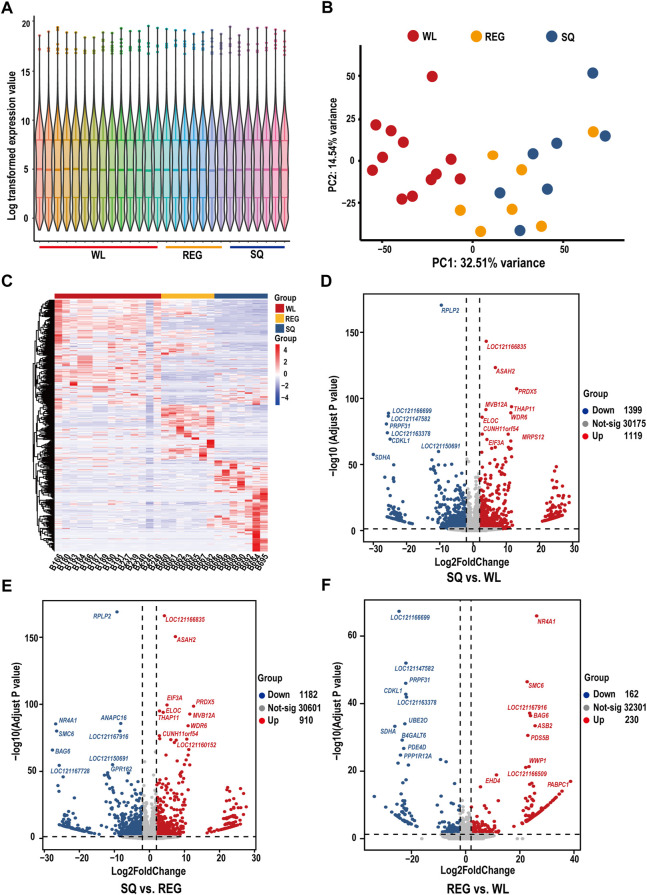
Expression levels, principal component analysis, differential gene expression heatmap and pair-to-pair-differential gene analysis based on the heart transcriptome of pikas. **(A)** Violin diagram of gene expression levels in the 28 samples. **(B)** PCA of the expression data of heart tissue samples from three populations of pikas. WL indicates Gansu pikas collected at 2,693 m, REG refers to plateau pikas collected at 3,406 m, and SQ refers to plateau pikas collected at 4,347 m. **(C)** Heatmap of the top 1% of genes according to their expression levels in 28 samples. **(D)** Volcano plot of differentially expressed genes in SQ vs. WL (note the ten most expressed genes). **(E)** Volcano plot of differentially expressed genes in SQ vs. REG (note the ten most expressed genes). **(F)** Volcano plot of differentially expressed genes in REG vs. WL (note the ten most expressed genes).

In the analysis of transcriptome expression results of different pika hearts, the results of PCA showed that WL samples were separated from REG and SQ samples and that there may be expression pattern differences between Gansu pika and plateau pika heart tissues (Figure 2B). According to a |log2FoldChange| ≥ 1 and padj < 0.05, 2518 DEGs were identified between plateau pikas from SQ and WL, and 1,119 genes were found to be significantly upregulated and 1,399 genes to be significantly downregulated (Figure 2D). Similarly, a total of 2093 DEGs were identified in the heart tissues of plateau pikas from REG vs. WL, among which 910 genes were significantly upregulated, and 1,182 genes were significantly downregulated (Figure 2F). On the other hand, only 392 DEGs were detected between the two populations of plateau pikas from SQ and REG, among which 230 genes were significantly upregulated, and 162 genes were significantly downregulated (Figure 2E). The results of volcano map analysis were consistent with the results of PCA, which showed significant differences in the expression patterns of cardiac tissues of different pikas. In other words, the expression patterns of plateau pikas and Gansu pikas were quite different. In addition, we found subtle differences among plateau pikas at different altitudes.

### Weighted gene co-expression network

**FIGURE 3:**
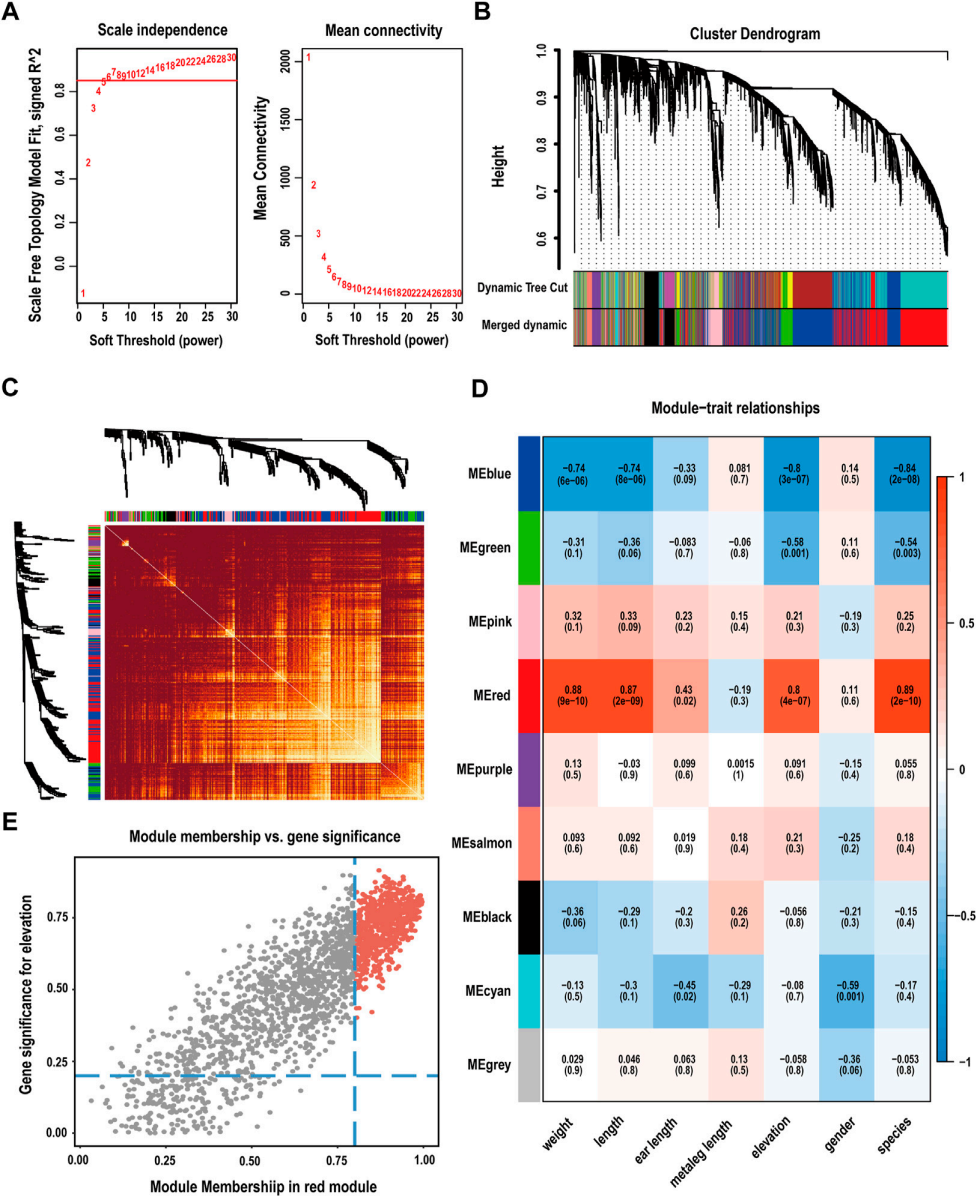
Construction of a weighted gene coexpression network. **(A)** Analysis of soft-thresholding power based on scale independence (left) and means connectivity (right). **(B)** Cluster dendrogram of genes. Modules considered to have high similarity were mixed. **(C)** Network heatmap plots of all genes in the WGCNA. **(D)** Heatmap of the correlations between modules and phenotypes. Altitude-related genes were mainly enriched in the red module. **(E)** Scatter plot of correlations between modules and phenotypes, screening of genes as hub genes according to |MM| > 0.8 and |GS| > 0.2 (red area).

In the rapid analysis of expression, 32,693 genes in 28 samples were quantitatively analysed. For a more precise analysis, we selected the top 20% of genes and eliminated outlier data to perform WGCNA. Module expression was summarized using the first principal component of gene expression for each module and regressed against the altitude of the sample. A soft threshold (*β* = 6, scale-free R^2^ = 0.85) was used to guarantee a scale-free network (Figure 3A; Supplementary Figure S3B), which identified thirteen modules and met the conditions required for standard scale-free network construction. By obtaining the feature vector of each module and merging similar modules, nine gene coexpression modules were obtained (Figures 3B,C). The number of genes per module ranged from a minimum of 10 (grey module) to a maximum of 2,457 (blue module) (Supplementary Tables S7, S8). Genes that could not be split into any modules were placed in the grey module and identified as non-coexpressed genes.

### Significantly correlated modules and enrichment of hub genes

To identify the modules that were most relevant to phenotype, we performed correlation analysis between modules and traits. The results showed that the red module (R = 0.8, P = 4E-0.7) presented the highest correlation with altitude and contained 2015 genes (Figure 3D; Supplementary Table S9). Hub genes are those with high connectivity in the network; these genes play crucial roles in biological processes and affect the regulation of other genes in related pathways. Using a kME value greater than 0.8 and gene and trait similarity values greater than 0.2 as screening criteria, 706 hub genes associated with high-altitude adaptability were screened from the red module (Figure 3E).

### Enrichment analysis of differentially expressed genes

**FIGURE 4:**
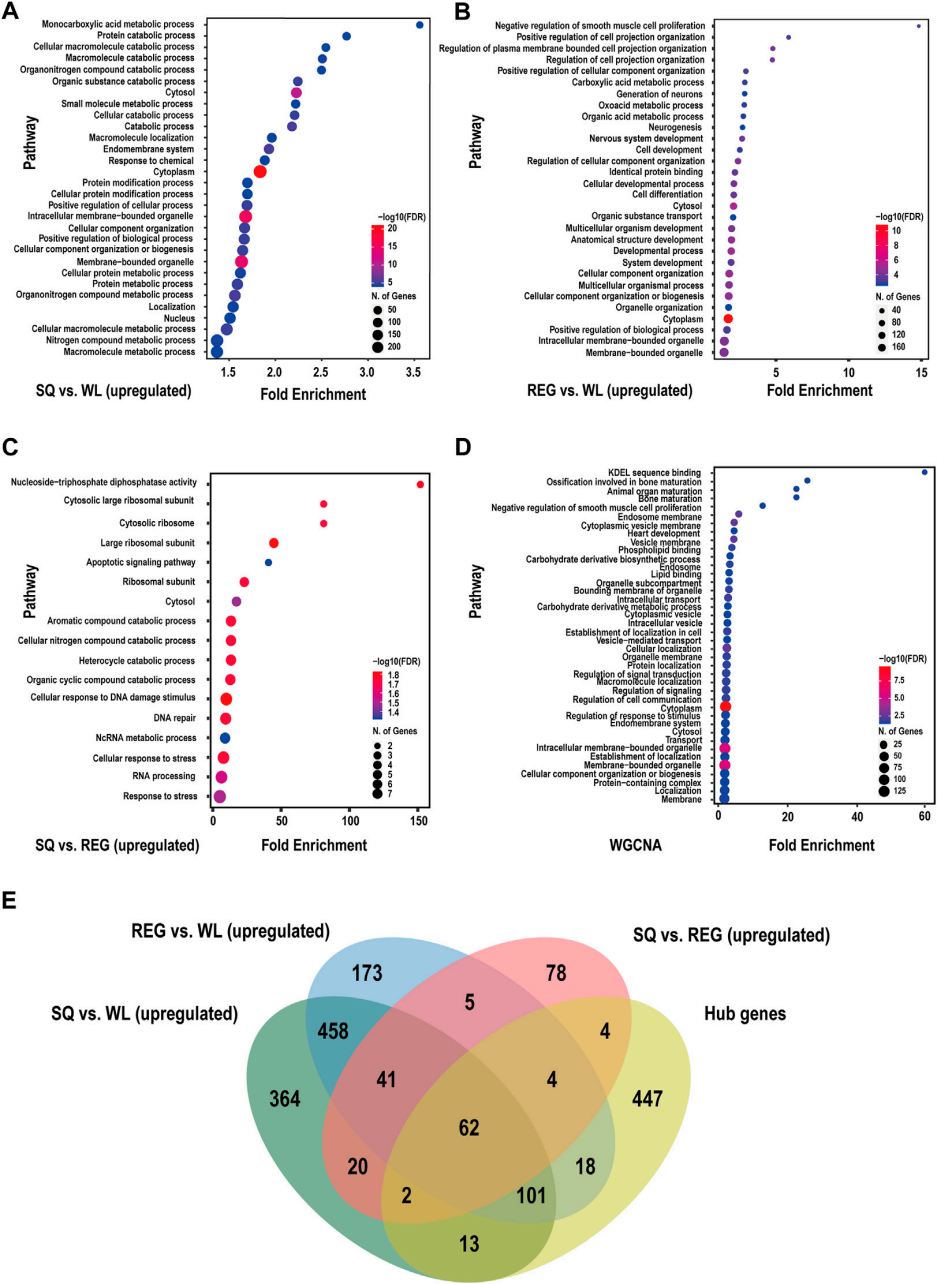
Functional enrichment analysis of DEGs (upregulated) and hub genes in plateau pika. **(A)** Functional analysis results of genes upregulated in SQ vs. WL. **(B)** Functional analysis results of genes upregulated in REG vs. WL. **(C)** Functional analysis results of genes upregulated in SQ vs. REG. **(D)** Functional analysis results of altitude-related hub genes screened by WGCNA. **(E)** Venn diagram presenting the results of interspecific and intraspecific variance analysis and WGCNA.

Through the differential expression analysis of WL, REG and SQ samples, we found significant differences between the plateau pika and Gansu pika, while only small differences were observed between plateau pikas from SQ and REG. GO enrichment analysis showed that compared with Gansu pikas from low altitudes, the upregulated genes identified in plateau pikas from SQ were mainly enriched in the monocarboxylic acid metabolic process, protein catabolic process, organonitrogen compound catabolic process, nitrogen compound metabolic process, positive regulation of biological process, response to chemical, positive regulation of cellular process, cellular component organization or biogenesis, endomembrane system and cellular component organization terms (Figure 4A; Supplementary Table S3). The downregulated genes identified in plateau pikas from SQ were mainly enriched in the regulation of signal transduction, cell communication, positive regulation of biological process, positive regulation of cellular process and intracellular membrane-bounded organelle terms (Supplementary Figure S4A; Supplementary Table S7).

When we compared differential gene expression between plateau pikas from REG and Gansu pikas, we found that the upregulated genes identified in plateau pikas were mainly enriched in the negative regulation of smooth muscle cell proliferation, positive regulation of cell projection organization, carboxylic acid metabolic process, oxoacid metabolic process, organic acid metabolic process, neurogenesis, nervous system development, regulation of cellular component organization, cell differentiation, system development, organic substance transport, and cellular developmental process categories (Figure 4B; Supplementary Table S4). The downregulated genes were mainly enriched in the regulation of cell communication, establishment of localization, regulation of signalling, negative regulation of biological process and negative regulation of cellular process categories (Supplementary Figure S4B; Supplementary Table S8).

There were 559 upregulated genes identified in SQ vs. WL and REG vs. WL (Figure 4E), and these upregulated genes were mainly enriched in the positive regulation of neuron projection development, nervous system development, cellular response to DNA damage stimulus, protein metabolic process, organonitrogen compound metabolic process and nitrogen compound metabolic process categories (Supplementary Figure S4F; Supplementary Table S6). There were 569 identical downregulated genes between plateau pikas and Gansu pikas at different altitudes (Supplementary Figure S4E). The enriched biological functions included phosphatidylinositol-mediated signalling, inositol lipid-mediated signalling, establishment of localization and cellular magnesium ion homeostasis (Supplementary Figure S4D; Supplementary Table S10).

Pairwise analysis of plateau pikas from different altitudes revealed that the upregulated genes of SQ samples were mainly enriched in the cellular nitrogen compound catabolic process, apoptosis signalling pathway, cellular response to DNA damage stimulus, DNA repair, and response to stress terms (Figure 4C; Supplementary Table S5). The downregulated genes identified in SQ samples were mainly enriched in the regulation of cell cycle, mitotic cell cycle, trans-Golgi network transport vesicle and microtubule cytoskeleton organization terms (Supplementary Figure S4D; Supplementary Table S9).

### Enrichment analysis of hub genes and related genes

To further investigate the biological functions of hub genes associated with altitude, we performed a GO term analysis (Figure 4B). Among the identified GO terms, the hub genes were mainly enriched in the intracellular membrane-bounded organelle, carbohydrate derivative biosynthetic process, phospholipid binding, lipid binding, cellular localization, regulation of signal transduction, ossification involved in bone maturation, animal organ maturation, regulation of signalling and heart development categories (Supplementary Table S6).

## Discussion

In this study, considering that gender differences will affect the results, we used the results of heart tissue expression of Gansu pika (including five females and nine males) to analyse the differences between different genders, and the results showed that there was no significant difference. Other transcription studies of pika heart and lung tissue have also not considered the effect of gender on the results (Zhang et al., 2022; Zhu et al., 2022). Therefore, instead of sex typing of plateau pikas and Gansu pikas, we directly analysed the transcriptome of heart tissues of 28 samples in order to explore the potential genetic mechanism of plateau pikas adapting to high altitude environment. We obtained normalized gene expression results for 32,693 genes in pika heart tissue. Then, we performed DEG and WGCNA to identify DEGs and their main functions. Our results showed that the plateau pika has adapted to the extreme environments of the QHTP *via* the protection of cardiomyocytes, tissue structure alterations and improvements in the blood circulation system and energy metabolism.

### Interspecies difference between *O. curzoniae* and *O. cansus*

Transcriptome analysis of the heart tissue of plateau pikas showed that the changes in several biological metabolic processes were significantly different from those in Gansu pikas. For example, the comparison of SQ and WL samples showed that upregulated genes were enriched in the monocarboxylic acid metabolism process, protein catabolism process, organic nitrogen compound catabolism process, and nitrogen compound metabolism process categories. The comparison of samples from REG vs. WL showed that upregulated genes were enriched in the carboxylic acid metabolism process, oxidative acid metabolism process, and organic acid metabolism process categories, indicating that plateau pikas have higher requirements for the Krebs cycle and energy metabolism than Gansu pikas. At high altitude, hypoxia causes a decrease in adenosine triphosphate (ATP), the formation of lactic acid, and alterations in many other metabolites (Cao et al., 2017). Chronic exposure to hypoxia induces adaptive cardiopulmonary changes to guarantee adequate oxygen intake and efficient delivery to tissues under conditions of limited oxygen utilization. Since oxygen is required in tissues to support oxidative metabolism, the process of adapting to hypoxia is likely to play a role the adjustment of relevant metabolic pathways and corresponding metabolites (Serkova et al., 2008). Studies on high-altitude adaptability have shown that the metabolic rates and body temperatures (Tb) of some species can be significantly reduced in response to cold or low-oxygen environments (Ostadal and Kolar, 2007). Hibernating animals show special adaptations in this regard, involving the reduction of their basal metabolic rate and energy consumption (Geiser, 2004; Long et al., 2005; Larson et al., 2014), and protection against ischaemic injury after cardiac arrest (Dave et al., 2006). Although the pika is a small mammal that does not hibernate, its central nervous system can perceive the downregulation of its metabolic rate and body temperature under hypoxia, and the pika can cope with challenges such as a high resting metabolic rate and low oxygen consumption rate through increases in the expression of thermogenic genes and energy metabolism (Wang et al., 2008). Therefore, to adapt to the extreme environment, plateau pikas require an efficient energy metabolism system to maintain the basic needs of life and compensatory mechanisms, such as the rational utilization of metabolic substrates, improvement of respiratory chain efficiency, and closer coupling of ATP supply and demand pathways. These findings are similar to those of studies of Tibetan Plateau aborigines showing that a major trend of plateau adaptation is an increased rate of evolution and positive selection for genes involved in energy metabolism (Simonson et al., 2010; Qiu et al., 2012; Qu et al., 2013b; Ai et al., 2014).

In addition to the differences in metabolic regulation identified between plateau pikas and Gansu pikas, neurogenesis, nervous system development, and system regulation were found to be enriched biological processes. Similarly, studies of the particular tolerance of Tibetans and Sherpas to hypoxia have found that Sherpas show downregulation of sympathetic nervous system (SNS) adrenergic receptors over time in response to hypoxic stimulation. Tibetans, on the other hand, show marked vagal innervation, which persists even after migration to lower elevations (Gilbert-Kawai et al., 2014; Gnecchi-Ruscone et al., 2018). Pichon et al. (2013) studied the cardiac adaptability of plateau pikas and found that the cardiac function of plateau pikas is affected by parasympathetic and sympathetic nerve regulation. Hypoxia can cause enhanced sympathetic nerve excitation, accelerate the heart rate, strengthen cardiac contraction, and improve cardiac output and blood oxygen transport capacity. These results are consistent with the results of the present study indicating that the cardiovascular adaptability of the plateau pika can be improved to cope with high-altitude environments *via* cardiac nerve regulation.

### Intraspecific differences in plateau pika

Pairwise analysis of plateau pikas from different altitudes showed that the DEGs of SQ samples were mainly enriched in the cellular response to DNA damage stimulus (*PAXX*, *MPG*, *ATM*, *NUDT1*, *ALKBH7* and *ZFYVE26*) and DNA repair (*PAXX*, *ATM*, *DCTPP1*, *MPG* and *ZFYVE26*) (Supplementary Table S5). Mammals living on the Tibetan Plateau face not only the harsh threat of hypoxia and cold but also apoptosis, DNA damage, inflammation and cancer caused by intense solar radiation and prolonged hypoxia (Scheinfeldt and Tishkoff, 2010; Bartels et al., 2013; Stoecklein et al., 2015). Dead or damaged cells release endogenous signals that activate inflammation to influence immune responses (Rock and Kono, 2008). Thereafter, the signals released by the damaged cells of eukaryotes are received, the expression of immune pathway components is activated, various cellular structural mechanisms are enhanced, and the bodily damage caused by hypoxia and ultraviolet radiation is resisted (Svobodová et al., 2012; Sklar et al., 2013).

In addition, the DEGs enriched in the above GO pathways (*NUDT1*, *PAXX*, *ATM* and *DCTPP1*) were highly expressed in SQ. PAXX nonhomologous end joining factor (*PAXX*) has been shown to be a key helper gene in nonhomologous end joining (NHEJ), the most prominent DNA double-strand break (DSB) repair pathway in mammalian cells, defining the molecular function of *PAXX* in KU accumulation at DNA ends (Liu et al., 2017). Ataxia-telangiectasia mutated (*ATM*) plays a key role in regulating the cellular response to ionizing radiation, and studies suggest that *ATM* may interact with, or be recruited to DNA-damaged chromatin (Andegeko et al., 2001; Bakkenist and Kastan., 2003). Nudix hydrolase 1 (*NUDT1*) and PAH-PASMCs hijack persistent oxidative stress and prevent the incorporation of oxidized nucleotides into DNA, thereby allowing cells to escape apoptosis, proliferate and to some extent participate in vascular remodelling in pulmonary hypertension (Mur et al., 2018; Vitry et al., 2021). A protein with NTP-PPase activity (*DCTPP1*) and the biological pathway in which it is involved (nucleoside triphosphate diphosphatase activity) were identified among the DEGs between samples from REG and SQ. This pathway can hydrolyse abnormal nucleotides in cells without affecting newly synthesized DNA or RNA, reduce the mutation rate, guarantee the stability of the genome, and play a “gatekeeping” role (Requena et al., 2014). Taken together, these results reflect the process of adaptation to hypoxia and UV radiation in plateau pikas at high altitudes. If the limits of respiratory motility, capillary uptake, and species transport have been reached, then maintaining homeostasis, improving metabolic efficiency, and immune regulation to reduce disease risk may be key to long-term adaptation to high altitudes. However, these aspects still need further study to elucidate and better understand the underlying mechanisms.

### Adaptive evolution of the heart at high altitude

Through the analysis of the cardiac transcriptome data of Gansu pikas and plateau pikas from SQ and REG, 2518, 2093 and 392 DEGs were obtained, and genes and pathways related to plateau adaptation were identified. These results proved that the heart tissue of plateau pikas shows gene expression changes induced by a high-altitude environment, similar to the results of other studies of plateau species adaptation. It is likely that these genes and tissues have been subject to natural selection and thus tend to confer beneficial adaptations to ongoing environmental pressure (Gilbert-Kawai et al., 2014).

In addition, we used WGCNA to identify modules with similar expression patterns, analyse the associations between modules and sample phenotypes (Pei et al., 2017), and comprehensively consider the related genes and pathways involved in high-altitude adaptation in heart tissue. Through the WGCNA method, we found one specific module related to high-altitude adaptation (Figure 3D, the red module). A total of 706 hub genes were screened from the red module (Figure 3E). GO enrichment analysis revealed some functions that were in accord with the results of DESeq2 differential expression analysis, such as the negative regulation of smooth muscle cell proliferation, regulation of signal transduction, intracellular transport, carbohydrate derivative biosynthetic process, and phospholipid binding (Figure 4D). These findings highlight that plateau pikas can adapt to extreme environments by upgrading material transport and metabolic pathways. Second, some of the identified genes (*DCHS1*, *PLXNB1* and *PHOSPHO1*) are involved in organ development, ossification and bone maturation as well as heart development (involving genes with *CASP7*, *NDRG4*, *IFT20*, *DCHS1*, *NDST1*, *RB1*, *SNX17*, *CDKN1B* and *MYL3*) and other biological functions (Supplementary Table S6). These are genes related to the maturation of heart organs and the formation of fibrous skeletal structures. For example, *DCHS1* plays an important role in mitral valve formation, and the mutation of this gene causes changes in the mitral valve similar to changes observed in human diseases (Durst et al., 2015). Similarly, to adapt to high-altitude environments, plateau yaks and Tibetan pigs have evolved larger heart tissues to provide greater blood oxygen delivery (Wang et al., 2020a; Ge et al., 2021; Tian et al., 2021; Wang et al., 2022). Pichon et al. (2013) identified left ventricular hypertrophy in pikas and showed that the heart exhibits increased angiogenesis mediated by the *VEGF* pathway. These results are similar to our findings and show that the heart of pikas has adaptively evolved under long-term exposure to high altitudes by strengthening the myocardial connective tissue band to provide structure and support to the heart, ensuring that the ventricle can drive blood flow and intermittently deliver blood to various parts of the body under high-altitude pressure (Saremi et al., 2017).

In this study, we revealed the genes and pathways related to cardiac adaptability through transcriptome analysis and assessed the basic characteristics of the pika cardiomyocyte population. However, it is still unknown how cardiac development and the regulation of myocardial components are controlled in pikas. Future studies combining single-cell transcriptomic and metabolic group analyses can shed further light on the evolutionary mechanism underlying the high-altitude adaptation of pika heart tissue.

## Conclusion

To explore the potential genetic mechanism underlying the high-altitude adaptation of pikas, we performed a comparative transcriptomic analysis of heart tissues of plateau pikas and Gansu pikas using the DESeq2 and WGCNA methods. We identified key genes and pathways related to high-altitude adaptation. In particular, these key genes are involved in cardiac organ maturation and the formation of fibrous skeletal structures (*DCHS1*, *PLXNB1*, *PHOSPHO1*) and in the response to hypoxia and ultraviolet radiation (*ATM*, *PAXX*, *MPG*, *NUDT1*, *ZFYVE26*). Moreover, *PDGF* family genes are involved in the regulation of the platelet-derived growth factor receptor signalling pathway (*PDGFRA* and *PBGFRB*). These results indicate that plateau pika cardiac structure and gene expression are regulated by natural selection in long-term high-altitude environments. Plateau pikas have adapted to extreme environments on the QHTP by enhancing the functional strength of the heart (myocardial connective tissue band, blood vessel wall, and blood supply capacity), maintaining the stability of the internal environment, and reducing bodily damage *via* immune regulation. Through interspecies difference analysis, we found that metabolic efficiency improvement and immune regulation to reduce disease risk may be critical to long-term adaptation to high-altitude environments. Our findings demonstrate the adaptability of the heart of plateau pikas to the extreme environments of the QHTP and provide new clues for further understanding the molecular mechanisms and characteristics of pika adaptation to high altitudes.

## Data Availability

The datasets presented in this study can be found in online repositories. The names of the repository/repositories and accession number(s) can be found below: https://db.cngb.org/search/project/CNP0003389/.

## References

[B1] Ai H. Yang B. Li J. Xie X. Chen H. Ren J. (2014). Population history and genomic signatures for high-altitude adaptation in Tibetan pigs. BMC Genomics 15. 10.1186/1471-2164-15-834 PMC4197311 25270331

[B2] Andegeko Y. Moyal L. Mittelman L. Tsarfaty I. Shiloh Y. Rotman G. (2001). Nuclear retention of ATM at sites of DNA double strand breaks. J. Biol. Chem. 276 (41), 38224–38230. 10.1074/jbc.M102986200 11454856

[B3] Andrews S. (2010). FastQC: A quality control tool for high throughput sequence data. Available at:http://www.bioinformatics.babraham.ac.uk/projects/fastqc .

[B4] Azad P. Stobdan T. Zhou D. Hartley I. Akbari A. Bafna V. (2017). High-altitude adaptation in humans: From genomics to integrative physiology. J. Mol. Med. 95 (12), 1269–1282. 10.1007/s00109-017-1584-7 28951950 PMC8936998

[B5] Bai K. He S. Shu L. Wang W. Lin S. Zhang Q. (2020). Identification of cancer stem cell characteristics in liver hepatocellular carcinoma by WGCNA analysis of transcriptome stemness index. Cancer Med. 9 (12), 4290–4298. 10.1002/cam4.3047 32311840 PMC7300398

[B6] Bakkenist C. J. Kastan M. B. (2003). DNA damage activates ATM through intermolecular autophosphorylation and dimer dissociation. Nature 421 (6922), 499–506. 10.1038/nature01368 12556884

[B7] Bao Q. Zhang X. Bao P. Liang C. Guo X. Chu M. (2021). Using weighted gene co-expression network analysis (WGCNA) to identify the hub genes related to hypoxic adaptation in yak (*Bos grunniens*). Genes Genomics 43 (10), 1231–1246. 10.1007/s13258-021-01137-5 34338989

[B8] Bartels K. Grenz A. Eltzschig H. K. (2013). Hypoxia and inflammation are two sides of the same coin. Proc. Natl. Acad. Sci. U. S. A. 110 (46), 18351–18352. 10.1073/pnas.1318345110 24187149 PMC3831992

[B9] Benjamini Y. Hochberg Y. (1995). Controlling the false discovery rate: A practical and powerful approach to multiple testing. J. R. Stat. Soc. Ser. B Methodol. 57 (1), 289–300. 10.1111/j.2517-6161.1995.tb02031.x

[B10] Bin Z. Steve H. (2005). A general framework for weighted gene co-expression network analysis. Stat. Appl. Genet. Mol. Biol. 4 (1), Article17. 10.2202/1544-6115.1128 16646834

[B11] Cao X. Bai Z. Ma L. Ma S. Ge R. (2017). Metabolic alterations of qinghai–tibet plateau pikas in adaptation to high altitude. High. Alt. Med. Biol. 18 (3), 219–225. 10.1089/ham.2016.0147 28846033

[B12] Chen S. Zhou Y. Chen Y. Gu J. (2018). fastp: an ultra-fast all-in-one FASTQ preprocessor. Bioinformatics 34 (17), i884–i890. 10.1093/bioinformatics/bty560 30423086 PMC6129281

[B13] Dave K. R. Prado R. Raval A. P. Drew K. L. Perez-Pinzon M. A. (2006). The arctic ground squirrel brain is resistant to injury from cardiac arrest during euthermia. Stroke 37 (5), 1261–1265. 10.1161/01.Str.0000217409.60731.38 16574920

[B14] Djan M. Stefanovic M. Velickovic N. Lavadinovic V. Alves P. C. Suchentrunk F. (2017). Brown hares (Lepus europaeus pallas, 1778) from the balkans: A refined phylogeographic model. Hystrix-Italian J. Mammal. 28 (2), 186–193. 10.4404/hystrix-28.2-12202

[B15] Dor Y. Camenisch T. D. Itin A. Fishman G. I. McDonald J. A. Carmeliet P. (2001). A novel role for VEGF in endocardial cushion formation and its potential contribution to congenital heart defects. Development 128 (9), 1531–1538. 10.1242/dev.128.9.1531 11290292

[B16] Durst R. Sauls K. Peal4 D. S. deVlaming A. Toomer K. Leyne M. (2015). Mutations in *DCHS1* cause mitral valve prolapse. Nature 525 (7567), 109–113. 10.1038/nature14670 26258302 PMC4720389

[B17] Feijo A. Ge D. Wen Z. Xia L. Yang Q. (2020). Divergent adaptations in resource-use traits explain how pikas thrive on the roof of the world. Funct. Ecol. 34 (9), 1826–1838. 10.1111/1365-2435.13609

[B18] Ge Q. Guo Y. Zheng W. Zhao S. Cai Y. Qi X. (2021). Molecular mechanisms detected in yak lung tissue via transcriptome-wide analysis provide insights into adaptation to high altitudes. Sci. Rep. 11 (1), 7786. 10.1038/s41598-021-87420-7 33833362 PMC8032655

[B19] Ge S. X. Jung D. Yao R. (2020). ShinyGO: A graphical gene-set enrichment tool for animals and plants. Bioinformatics 36 (8), 2628–2629. 10.1093/bioinformatics/btz931 31882993 PMC7178415

[B20] Geiser F. (2004). Metabolic rate and body temperature reduction during hibernation and daily torpor. Annu. Rev. Physiol. 66 (1), 239–274. 10.1146/annurev.physiol.66.032102.115105 14977403

[B21] Gilbert-Kawai E. T. Milledge J. S. Grocott M. P. W. Martin D. S. (2014). King of the mountains: Tibetan and sherpa physiological adaptations for life at high altitude. Physiol. (Bethesda) 29 (6), 388–402. 10.1152/physiol.00018.2014 25362633

[B22] Gnecchi-Ruscone G. A. Abondio P. Fanti S. D. Sarno S. Sherpa M. G. Sherpa P. T. (2018). Evidence of polygenic adaptation to high altitude from Tibetan and Sherpa genomes. Genome Biol. Evol. 10 (11), 2919–2930. 10.1093/gbe/evy233 30335146 PMC6239493

[B23] Gong G. Fan Y. Yan X. Li W. Yan X. Liu H. (2022). Identification of genes related to hair follicle cycle development in inner Mongolia cashmere goat by WGCNA. Front. Vet. Sci. 9, 894380. 10.3389/fvets.2022.894380 35774980 PMC9237575

[B24] Guan J. Long K. Ma J. Zhang J. He D. Jin L. (2017). Comparative analysis of the microRNA transcriptome between yak and cattle provides insight into high-altitude adaptation. PeerJ 5, e3959. 10.7717/peerj.3959 29109913 PMC5671665

[B25] Gureev A. A. (1964). The phylogeny of the hares, rabbits and pikas (Lagomorpha Mammalia), in the light of new data paleontology and comparative morphology. Doklady Proc. Acad. Sci. USSR Biol. Sci. Sect. 155, 319–321.

[B26] Hao Y. Xiong Y. Cheng Y. Song G. Jia C. Qu Y. (2019). Comparative transcriptomics of 3 high-altitude passerine birds and their low-altitude relatives. Proc. Natl. Acad. Sci. U. S. A. 116 (24), 11851–11856. 10.1073/pnas.1819657116 31127049 PMC6576129

[B27] Jukes E. (2018). Lagomorphs: Pikas, rabbits and hares of the world. Ref. Rev. 32 (6), 25–27. 10.1108/rr-05-2018-0082

[B28] Koju N. P. He K. Chalise M. K. Ray C. Chen Z. Zhang B. (2017). Multilocus approaches reveal underestimated species diversity and inter-specific gene flow in pikas (*Ochotona*) from southwestern China. Mol. Phylogenet. Evol. 107, 239–245. 10.1016/j.ympev.2016.11.005 27838310

[B29] Kucukural A. Yukselen O. Ozata D. M. Moore M. J. Garber M. (2019). DEBrowser: Interactive differential expression analysis and visualization tool for count data. BMC Genomics 20 (1), 6. 10.1186/s12864-018-5362-x 30611200 PMC6321710

[B30] Lan D. Xiong X. Ji W. Li J. Mipam T. D. Ai Y. (2018). Transcriptome profile and unique genetic evolution of positively selected genes in yak lungs. Genetica 146 (2), 151–160. 10.1007/s10709-017-0005-8 29285685

[B31] Langfelder P. Horvath S. (2008). Wgcna: an R package for weighted correlation network analysis. BMC Bioinforma. 9, 559. 10.1186/1471-2105-9-559 PMC2631488 19114008

[B32] Larson J. Drew K. L. Folkow L. P. Milton S. L. Park T. J. (2014). No oxygen? No problem! Intrinsic brain tolerance to hypoxia in vertebrates. J. Exp. Biol. 217 (7), 1024–1039. 10.1242/jeb.085381 24671961 PMC3966918

[B33] Liu J. Wu Z. Sun R. Nie S. Meng H. Zhong Y. (2020). Using mRNAsi to identify prognostic-related genes in endometrial carcinoma based on WGCNA. Life Sci. 258, 118231. 10.1016/j.lfs.2020.118231 32791150

[B34] Liu M. Qu J. Wang Z. Wang Y. Zhang Y. Zhang Z. (2012). Behavioral mechanisms of male sterilization on plateau pika in the Qinghai-Tibet plateau. Behav. Process. 89 (3), 278–285. 10.1016/j.beproc.2011.12.009 22206991

[B35] Liu X. Shao Z. Jiang W. Lee B. J. Zha S. (2017). PAXX promotes KU accumulation at DNA breaks and is essential for end-joining in XLF-deficient mice. Nat. Commun. 8, 13816. 10.1038/ncomms13816 28051062 PMC5216128

[B36] Long M. Y. Xiongwei Z. Rivera P. M. Øivind T. Barnes B. M. LaManna J. C. (2005). Absence of cellular stress in brain after hypoxia induced by arousal from hibernation in Arctic ground squirrels. Am. J. Physiol. Regul. Integr. Comp. Physiol. 289 (5), R1297–R1306. 10.1152/ajpregu.00260.2005 15976308

[B37] Love M. I. Huber W. Anders S. (2014). Moderated estimation of fold change and dispersion for RNA-seq data with DESeq2. Genome Biol. 15 (12), 550. 10.1186/s13059-014-0550-8 25516281 PMC4302049

[B38] Marfell B. J. O’Brien R. Griffin J. F. T. (2013). Global gene expression profiling of monocyte-derived macrophages from red deer (*Cervus elaphus*) genotypically resistant or susceptible to *Mycobacterium avium* subspecies paratuberculosis infection. Dev. Comp. Immunol. 40 (2), 210–217. 10.1016/j.dci.2013.02.004 23454067

[B39] Maurano M. T. Humbert R. Rynes E. Thurman R. E. Haugen E. Wang H. (2012). Systematic localization of common disease-associated variation in regulatory DNA. Science 337 (6099), 1190–1195. 10.1126/science.1222794 22955828 PMC3771521

[B40] Melo Ferreira J. de Matos A. L. Areal H. Lissovsky A. A. Carneiro M. Esteves P. J. (2015). The phylogeny of pikas (Ochotona) inferred from a multilocus coalescent approach. Mol. Phylogenet. Evol. 84, 240–244. 10.1016/j.ympev.2015.01.004 25637497

[B41] Montuire S. (2001). “Lagomorpha rabbits, hares and pikas,” in Encyclopedia of life sciences. New York, NY: John Wiley & Sons, Ltd.

[B42] Mur P. Jemth A. S. Bevc L. Amaral N. Navarro M. Valdes-Mas R. (2018). Germline variation in the oxidative DNA repair genes NUDT1 and OGG1 is not associated with hereditary colorectal cancer or polyposis. Hum. Mutat. 39 (9), 1214–1225. 10.1002/humu.23564 29900613

[B43] Ostadal B. Kolar F. (2007). Cardiac adaptation to chronic high-altitude hypoxia: Beneficial and adverse effects. Respir. Physiol. Neurobiol. 158 (2-3), 224–236. 10.1016/j.resp.2007.03.005 17442631

[B44] Patro R. Duggal G. Love M. I. Irizarry R. A. Kingsford C. (2017). Salmon provides fast and bias-aware quantification of transcript expression. Nat. Methods 14 (4), 417–419. 10.1038/nmeth.4197 28263959 PMC5600148

[B45] Pei G. Chen L. Zhang W. (2017). WGCNA application to proteomic and metabolomic data analysis. Methods Enzymol. 585, 135–158. 10.1016/bs.mie.2016.09.016 28109426

[B46] Pichon A. e. Voituron N. Bai Z. Jeton F. Tana W. Marchant D. (2015). Comparative ventilatory strategies of acclimated rats and burrowing plateau pika (*Ochotona curzoniae*) in response to hypoxic-hypercapnia. Comp. Biochem. Physiol. A Mol. Integr. Physiol. 187, 103–110. 10.1016/j.cbpa.2015.05.004 25988712

[B47] Pichon A. Zhenzhong B. Marchant D. Jin G. Voituron N. Haixia Y. (2013). Cardiac adaptation to high altitude in the plateau pika (*Ochotona curzoniae*). Physiol. Rep. 1 (2), e00032. 10.1002/phy2.32 24303117 PMC3831927

[B48] Qi X. Wang X. Zhu S. Rao X. Wei L. Wei D. (2008). Hypoxic adaptation of the hearts of plateau zokor (Myospalax baileyi) and plateau pika (Ochotona curzoniae). Sheng Li Xue Bao 60 (3), 348–354. 18560725

[B49] Qiu Q. Zhang G. Ma T. Qian W. Wang J. Ye Z. (2012). The yak genome and adaptation to life at high altitude. Nat. Genet. 44 (8), 946–949. 10.1038/ng.2343 22751099

[B50] Qu J. Li W. Yang M. Ji W. Zhang Y. (2013a). Life history of the plateau pika (*Ochotona curzoniae*) in alpine meadows of the Tibetan Plateau. Mamm. Biol. 78 (1), 68–72. 10.1016/j.mambio.2012.09.005

[B51] Qu Y. Zhao H. Han N. Zhou G. Song G. Gao B. (2013b). Ground tit genome reveals avian adaptation to living at high altitudes in the Tibetan plateau. Nat. Commun. 4, 2071. 10.1038/ncomms3071 23817352

[B52] Ream M. Ray A. M. Chandra R. Chikaraishi D. M. (2008). Early fetal hypoxia leads to growth restriction and myocardial thinning. Am. J. Physiol. Regul. Integr. Comp. Physiol. 295 (2), R583–R595. 10.1152/ajpregu.00771.2007 18509101 PMC2519936

[B53] Requena C. E. Pérez-Moreno G. Ruiz-Pérez L. M. Vidal A. E. González-Pacanowska D. (2014). The NTP pyrophosphatase DCTPP1 contributes to the homoeostasis and cleansing of the dNTP pool in human cells. Biochem. J. 459 (1), 171–180. 10.1042/BJ20130894 24467396

[B54] Rock K. L. Kono H. (2008). The inflammatory response to cell death. Annu. Rev. Pathol. 3 (1), 99–126. 10.1146/annurev.pathmechdis.3.121806.151456 18039143 PMC3094097

[B55] Sahota I. S. Panwar N. S. (2013). Prevalence of chronic mountain sickness in high altitude districts of Himachal Pradesh. Indian J. Occup. Environ. Med. 17 (3), 94–100. 10.4103/0019-5278.130839 24872667 PMC4035612

[B56] Saremi F. Sánchez-Quintana D. Mori S. Muresian H. Spicer D. E. Hassani C. (2017). Fibrous skeleton of the heart: Anatomic overview and evaluation of pathologic conditions with CT and MR imaging. Radiographics 37 (5), 1330–1351. 10.1148/rg.2017170004 28820653

[B57] Scheinfeldt L. B. Tishkoff S. A. (2010). Living the high life: High-altitude adaptation. Genome Biol. 11 (9), 133–3. 10.1186/gb-2010-11-9-133 20979669 PMC2965377

[B58] Serkova N. J. Reisdorph N. A. Tissot van Patot M. C. (2008). Metabolic markers of hypoxia: Systems biology application in biomedicine. Toxicol. Mech. Methods 18 (1), 81–95. 10.1080/15376510701795769 20020894

[B59] Simonson T. S. Yang Y. Huff C. D. Yun H. Qin G. Witherspoon D. J. (2010). Genetic evidence for high-altitude adaptation in tibet. Science 329 (5987), 72–75. 10.1126/science.1189406 20466884

[B60] Sklar L. R. Almutawa F. Lim H. W. Hamzavi I. (2013). Effects of ultraviolet radiation, visible light, and infrared radiation on erythema and pigmentation: A review. Photochem. Photobiol. Sci. 12 (1), 54–64. 10.1039/c2pp25152c 23111621

[B61] Smith A. T. BadingqiuyingWilson M. C. Hogan B. W. (2019). Functional-trait ecology of the plateau pika *Ochotona curzoniae* in the Qinghai-Tibetan Plateau ecosystem. Integr. Zool. 14 (1), 87–103. 10.1111/1749-4877.12300 29316275

[B62] Smith A. T. Foggin J. M. (1999). The plateau pika (*Ochotona curzoniae*) is a keystone species for biodiversity on the Tibetan plateau. Anim. Conserv. 2 (4), 235–240. 10.1111/j.1469-1795.1999.tb00069.x

[B63] Solari K. A. Hadly E. A. (2018). Evolution for extreme living: Variation in mitochondrial cytochrome c oxidase genes correlated with elevation in pikas (genus ochotona). Integr. Zool. 13 (5), 517–535. 10.1111/1749-4877.12332 29851233

[B64] Stoecklein V. M. Osuka A. Ishikawa S. Lederer M. R. Wanke-Jellinek L. Lederer J. A. (2015). Radiation exposure induces inflammasome pathway activation in immune cells. J. Immunol. 194 (3), 1178–1189. 10.4049/jimmunol.1303051 25539818 PMC4326002

[B65] Storey J. D. (2002). A direct approach to false discovery rates. J. R. Stat. Soc. Ser. B Stat. Methodol. 64 (3), 479–498. 10.1111/1467-9868.00346

[B66] Storey J. D. Taylor J. E. Siegmund D. (2004). Strong control, conservative point estimation and simultaneous conservative consistency of false discovery rates: A unified approach. J. R. Stat. Soc. B 66 (1), 187–205. 10.1111/j.1467-9868.2004.00439.x

[B67] Su J. Lian X. Zhang T. Cui Q. Liu J. (2005). Hay-pile caches as winter food by Gansu Pikas and its biological signif icance. Acta Theriol. Sin. 24 (1), 23.

[B68] Su J. Liu J. (2000). Overwinter of small herbivorous mammals inhabiting Alpine area. Acta Theriol. Sin. 20 (3), 186–192.

[B69] Svobodová A. R. Galandáková A. Šianská J. Doležal D. Lichnovská R. Ulrichová J. (2012). DNA damage after acute exposure of mice skin to physiological doses of UVB and UVA light. Arch. Dermatol. Res. 304 (5), 407–412. 10.1007/s00403-012-1212-x 22271212

[B70] Tang J. Kong D. Cui Q. Wang K. Zhang D. Gong Y. (2018). Prognostic genes of breast cancer identified by gene co-expression network analysis. Front. Oncol. 8, 374. 10.3389/fonc.2018.00374 30254986 PMC6141856

[B71] Tang Q. Gu Y. Zhou X. Jin L. Guan J. Liu R. (2017). Comparative transcriptomics of 5 high-altitude vertebrates and their low-altitude relatives. Gigascience 6 (12), 1–9. 10.1093/gigascience/gix105 PMC5729692 29149296

[B72] Tian X. Ma J. Wu Y. Zhang P. Li Q. Zhang H. (2021). Functional analysis of the brain natriuretic peptide gene for high-altitude adaptation in Tibetan pigs. Gene 768, 145305. 10.1016/j.gene.2020.145305 33186614

[B73] Vitry G. Paulin R. Grobs Y. Lampron M.-C. Shimauchi T. Lemay S.-E. (2021). Oxidized DNA precursors cleanup by NUDT1 contributes to vascular remodeling in pulmonary arterial hypertension. Am. J. Respir. Crit. Care Med. 203 (5), 614–627. 10.1164/rccm.202003-0627OC 33021405

[B74] Wang C. Zhao X. Liu Z. Lippert P. C. Graham S. A. Coe R. S. (2008). Constraints on the early uplift history of the Tibetan Plateau. Proc. Natl. Acad. Sci. U. S. A. 105 (13), 4987–4992. 10.1073/pnas.0703595105 18362353 PMC2278176

[B75] Wang D. Sun R. Wang Z. (1999). Effects of photoperiod and temperature on browh adipose tissue thermogenic properties in plateau pika. Zoological Res. 20 (5), 347–351.

[B76] Wang H. Wang X. Li M. Wang S. Chen Q. Lu S. (2022). Identification of key sex-specific pathways and genes in the subcutaneous adipose tissue from pigs using WGCNA method. BMC Genom. Data 23 (1), 35. 10.1186/s12863-022-01054-w 35538407 PMC9086418

[B77] Wang J. Zhang Y. Wang D. (2006). Seasonal thermogenesis and body mass regulation in plateau pikas (*Ochotona curzoniae*). Oecologia 149 (3), 373–382. 10.1007/s00442-006-0469-1 16823564

[B78] Wang Q. Li D. Guo A. Li M. Li L. Zhou J. (2020a). Whole-genome resequencing of Dulong Chicken reveal signatures of selection. Br. Poult. Sci. 61 (6), 624–631. 10.1080/00071668.2020.1792832 32627575

[B79] Wang X. Liang D. Jin W. Tang M. Liu S. Zhang P. (2020b). Out of tibet: Genomic perspectives on the evolutionary history of extant pikas. Mol. Biol. Evol. 37 (6), 1577–1592. 10.1093/molbev/msaa026 32027372

[B80] Wilson M. C. Smith A. T. (2015). The pika and the watershed: The impact of small mammal poisoning on the ecohydrology of the Qinghai-Tibetan Plateau. Ambio 44 (1), 16–22. 10.1007/s13280-014-0568-x 25331028 PMC4293360

[B81] Wu Y. Cheng T. Liu C. Liu D. Zhang Q. Long R. (2016). Systematic identification and characterization of long non-coding RNAs in the silkworm, *Bombyx mori* . PloS One 11 (1), e0147147. 10.1371/journal.pone.0147147 26771876 PMC4714849

[B82] Yang J. Chen C. Jin X. Liu L. Lin J. Kang X. (2021). Wfs1 and related molecules as key candidate genes in the Hippocampus of depression. Front. Genet. 11, 589370. 10.3389/fgene.2020.589370 33552119 PMC7863986

[B83] Zhang B. Chamba Y. Shang P. Wang Z. Ma J. Wang L. (2017). Comparative transcriptomic and proteomic analyses provide insights into the key genes involved in high-altitude adaptation in the Tibetan pig. Sci. Rep. 7 (1), 3654. 10.1038/s41598-017-03976-3 28623314 PMC5473931

[B84] Zhang X.-Z. Fu L. Zou X.-Y. Li S. Ma X.-D. Xie L. (2022). Lung transcriptome analysis for the identification of genes involved in the hypoxic adaptation of plateau pika (Ochotona curzoniae). Comp. Biochem. Physiol. Part D. Genomics Proteomics 41, 100943. 10.1016/j.cbd.2021.100943 34861554

[B85] Zhu H. Zhong L. Li J. Wang S. Qu J. (2021). Differential expression of metabolism-related genes in plateau pika (*Ochotona curzoniae*) at different altitudes of Qinghai–Tibet Plateau. Front. Genet. 12, 784811. 10.3389/fgene.2021.784811 35126457 PMC8811202

[B86] Zhu H. Zhong L. Li J. Wang S. Qu J. (2022). Differential expression of metabolism-related genes in plateau pika (ochotona curzoniae) at different altitudes on the Qinghai-Tibet Plateau. Front. Genet. 12, 784811. 10.3389/fgene.2021.784811 35126457 PMC8811202

[B87] Zhu M. Xie H. Wei X. Dossa K. Yu Y. Hui S. (2019). WGCNA analysis of salt-responsive core transcriptome identifies novel hub genes in rice. Genes 10 (9), E719. 10.3390/genes10090719 PMC6771013 31533315

[B88] Zhu W. Hou D. Sun S. Wang Z. (2017). White adipose tissue undergoes ‘browning’in tree shrews (*Tupaia belangeri*) during cold acclimation. Mamm. Study 42 (4), 1–8. 10.3106/041.042.0405

