# Supplementary material for: Transcriptome analysis of pika heart tissue reveals mechanisms underlying the adaptation of a keystone species on the roof of the world
Source: Front Genet. 2022 Nov 23;13:1020789. doi: 10.3389/fgene.2022.1020789 (PMC9728954; doi:10.3389/fgene.2022.1020789)
Supplement: Supplementary file 1 [file DataSheet1.PDF]

Supplementary Material

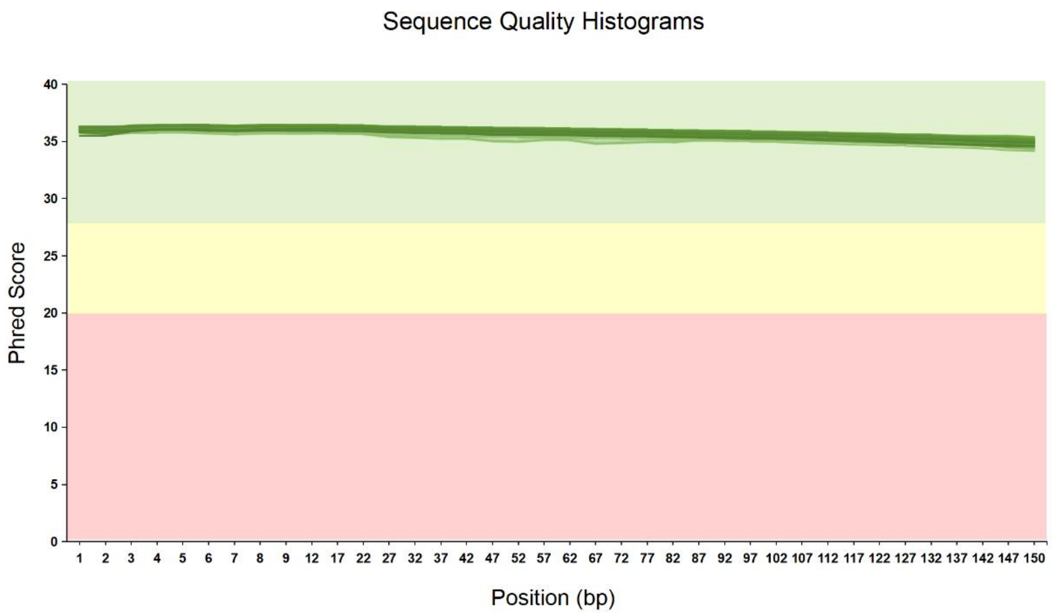

Supplementary Figure 1 Sequence Quality Histograms. The mean quality value across each base position in the read. The green range indicates good data quality, the yellow range indicates good data quality, and the red range indicates poor data quality.

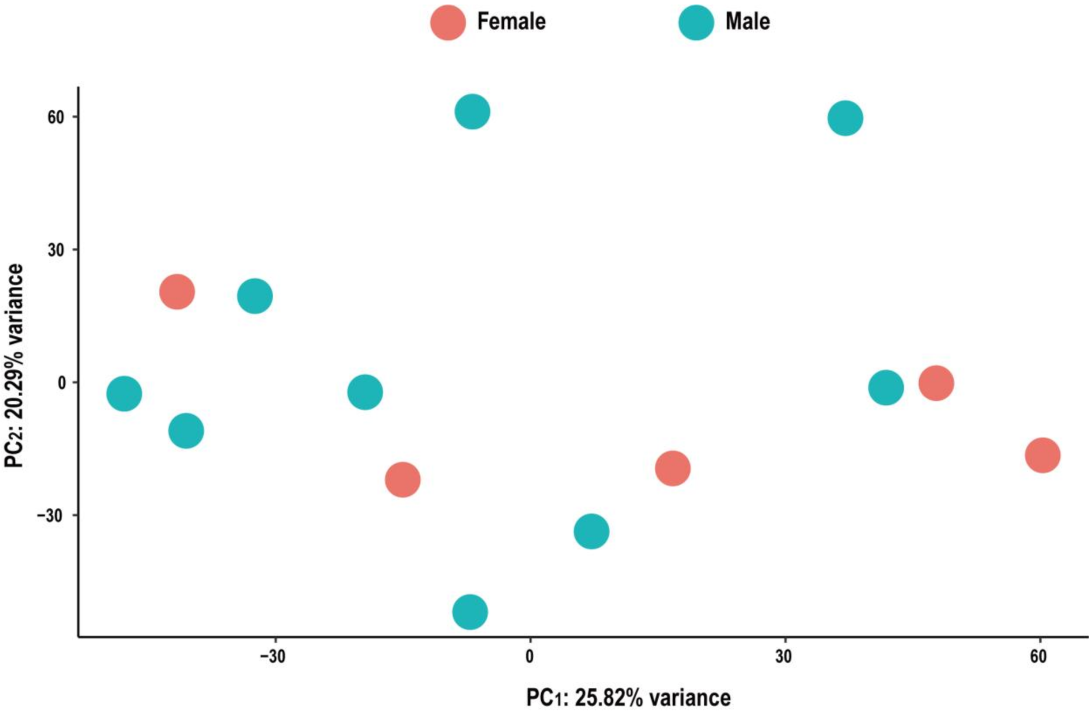

Supplementary Figure 2 PCA plotting on the overall transcriptomic differences of heart tissue from male and female of *O. cansus*.

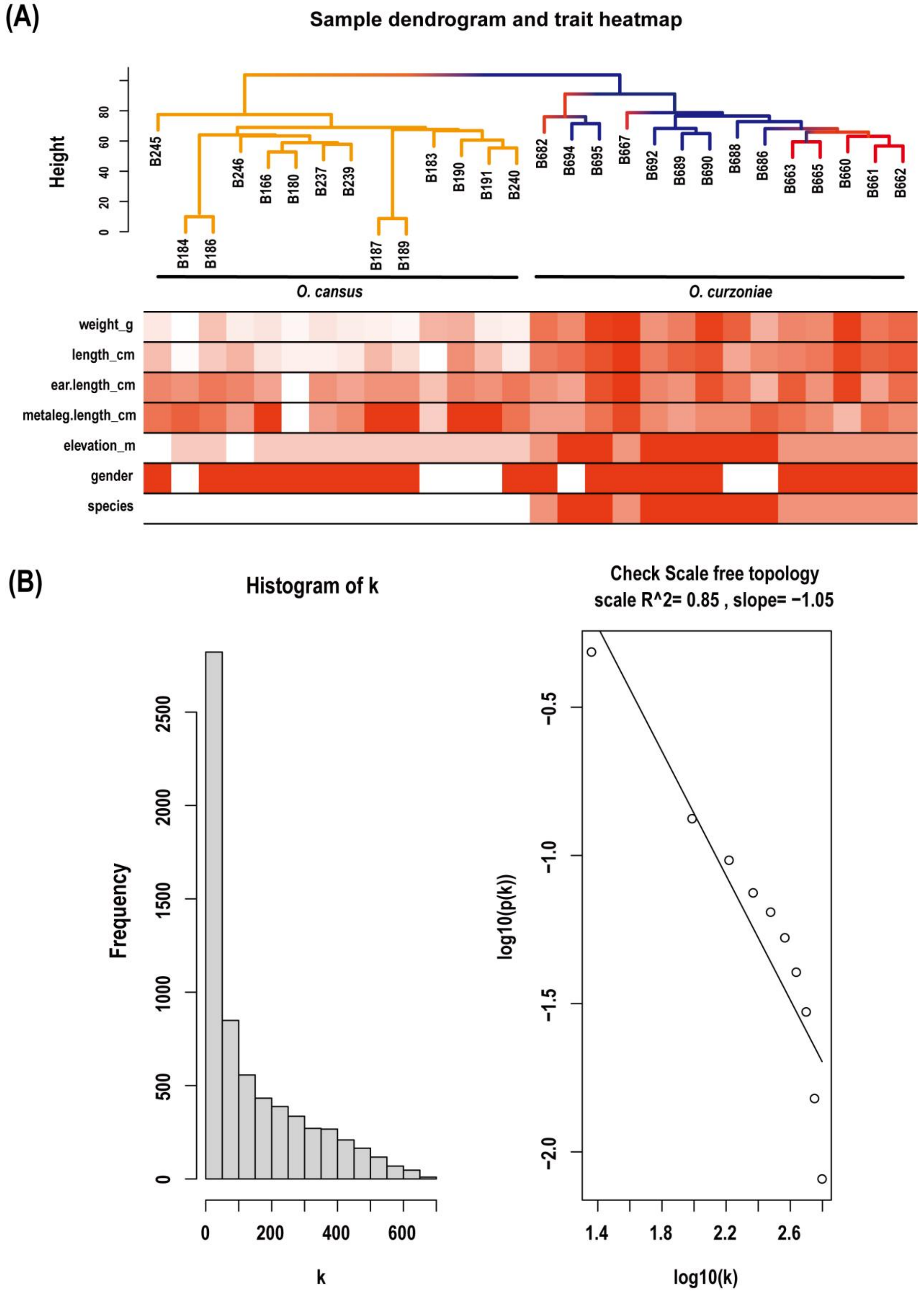

Supplementary Figure 3 (A) Clustering dendrogram of 28 samples. **(B)** Histogram and check scale free topology.

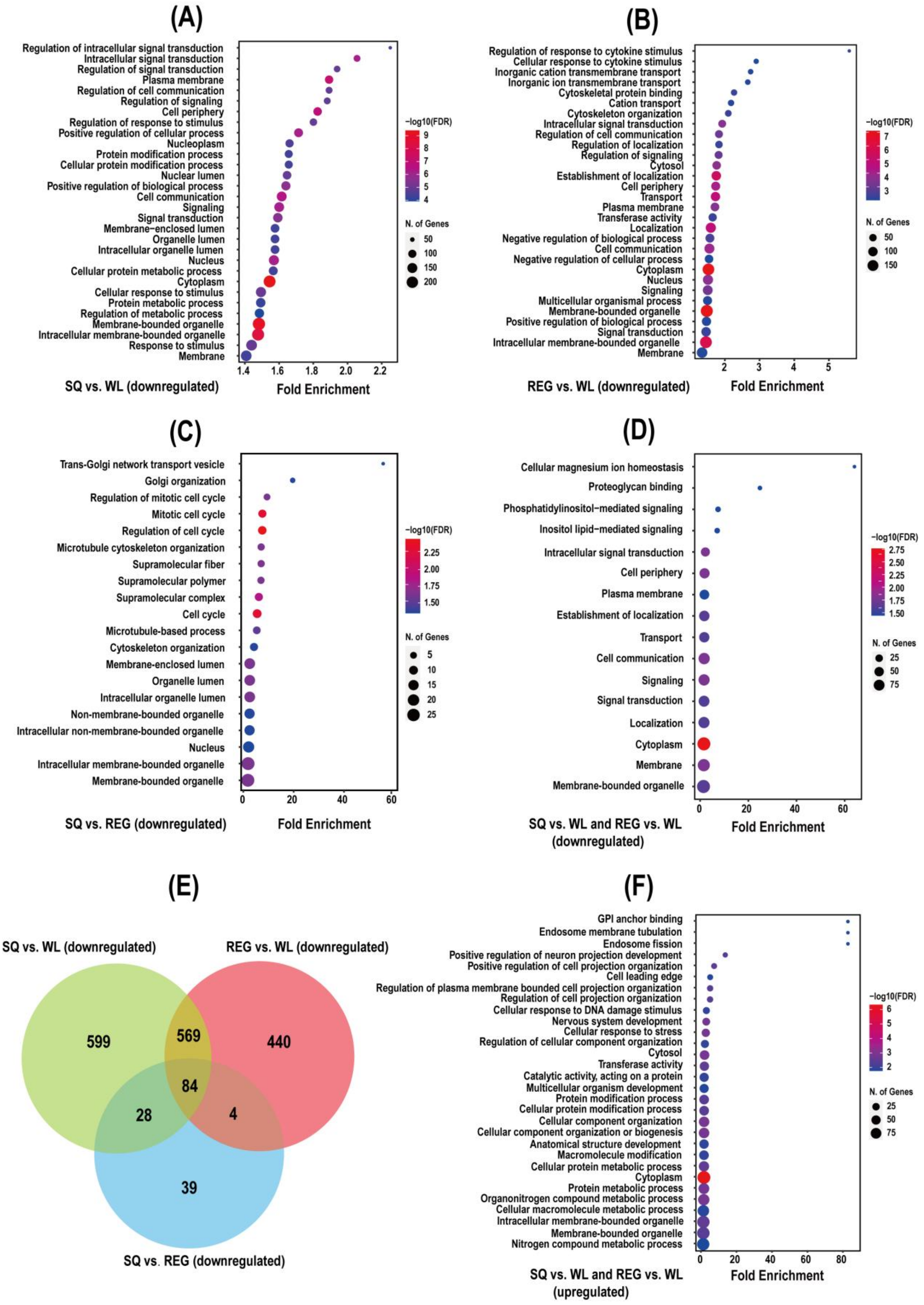

Supplementary Figure 4 (A) Functional analysis results of genes downregulated in SQ vs. WL. (B) Functional analysis results of genes downregulated in REG vs. WL. (C) Functional analysis results of genes downregulated in SQ vs. REG. (D) Functional analysis of down-regulation of genes identical to SQ vs. WL and REG vs. WL. (E) The Venn diagram of downregulated genes was screened by differential analysis. (F) Functional analysis of upregulated of genes identical to SQ vs. WL and REG vs. WL.

Supplementary Table 1 Collection information about specimens.

Acquisition number	Sample name	Gender	Museums	Localities	Latitude	Longitude	Elevation
sch2021166	*O. cansus*	male	IOZCAS	China, Sichuan, Wanglang National Nature Reserve	32.9050	104.0540	2963 m
sch2021180	*O. cansus*	male	IOZCAS	China, Sichuan, Wanglang National Nature Reserve	32.9050	104.0540	2963 m
sch2021183	*O. cansus*	female	IOZCAS	China, Sichuan, Wanglang National Nature Reserve	32.9050	104.0540	2963 m
sch2021184	*O. cansus*	female	IOZCAS	China, Sichuan, Wanglang National Nature Reserve	32.9050	104.0540	2963 m
sch2021186	*O. cansus*	female	IOZCAS	China, Sichuan, Wanglang National Nature Reserve	32.9050	104.0540	2963 m
sch2021187	*O. cansus*	male	IOZCAS	China, Sichuan, Wanglang National Nature Reserve	32.9050	104.0540	2963 m
sch2021189	*O. cansus*	male	IOZCAS	China, Sichuan, Wanglang National Nature Reserve	32.9050	104.0540	2963 m
sch2021190	*O. cansus*	female	IOZCAS	China, Sichuan, Wanglang National Nature Reserve	32.9050	104.0540	2963 m
sch2021191	*O. cansus*	female	IOZCAS	China, Sichuan, Wanglang National Nature Reserve	32.9050	104.0540	2963 m
sch2021237	*O. cansus*	male	IOZCAS	China, Sichuan, Wanglang National Nature Reserve	32.9050	104.0540	2963 m
sch2021239	*O. cansus*	male	IOZCAS	China, Sichuan, Wanglang National Nature Reserve	32.9050	104.0540	2963 m
sch2021240	*O. cansus*	male	IOZCAS	China, Sichuan, Wanglang National Nature Reserve	32.9050	104.0540	2963 m
sch2021245	*O. cansus*	male	IOZCAS	China, Sichuan, Wanglang National Nature Reserve	32.9664	104.1158	2490 m
sch2021246	*O. cansus*	male	IOZCAS	China, Sichuan, Wanglang National Nature Reserve	32.9664	104.1158	2490 m
sch2021660	*O. curzoniae*	male	IOZCAS	China, Sichuan, Zoige Wetland National Nature Reserve	33.6499	102.8204	3406 m
sch2021661	*O. curzoniae*	male	IOZCAS	China, Sichuan, Zoige Wetland National Nature Reserve	33.6499	102.8204	3406 m
sch2021662	*O. curzoniae*	male	IOZCAS	China, Sichuan, Zoige Wetland National Nature Reserve	33.6499	102.8204	3406 m
sch2021663	*O. curzoniae*	female	IOZCAS	China, Sichuan, Zoige Wetland National Nature Reserve	33.6499	102.8204	3406 m
sch2021665	*O. curzoniae*	female	IOZCAS	China, Sichuan, Zoige Wetland National Nature Reserve	33.6499	102.8204	3406 m
sch2021667	*O. curzoniae*	male	IOZCAS	China, Sichuan, Zoige Wetland National Nature Reserve	33.6499	102.8204	3406 m
sch2021682	*O. curzoniae*	male	IOZCAS	China, Sichuan, Zoige Wetland National Nature Reserve	33.6499	102.8204	3406 m

Acquisition number	Sample name	Gender	Museums	Localities	Latitude	Longitude	Elevation
sch2021686	*O. curzoniae*	female	IOZCAS	China, Sichuan, Shiqu	32.9952	98.4415	4347 m
sch2021688	*O. curzoniae*	female	IOZCAS	China, Sichuan, Shiqu	32.9952	98.4415	4347 m
sch2021689	*O. curzoniae*	male	IOZCAS	China, Sichuan, Shiqu	32.9952	98.4415	4347 m
sch2021690	*O. curzoniae*	male	IOZCAS	China, Sichuan, Shiqu	32.9952	98.4415	4347 m
sch2021692	*O. curzoniae*	female	IOZCAS	China, Sichuan, Shiqu	32.9952	98.4415	4347 m
sch2021694	*O. curzoniae*	female	IOZCAS	China, Sichuan, Shiqu	32.9952	98.4415	4347 m
sch2021695	*O. curzoniae*	male	IOZCAS	China, Sichuan, Shiqu	32.9952	98.4415	4347 m

* Voucher specemens were preserved in the National Zoological Museum, Institute of Zoology, Chinese Academy of Sciences. (IOZCAS)

Supplementary Table 2 Basic information of transcriptome sequencing and alignment rates.

Species	Field code	Acquisition number	ID	Clean Reads (bp)	Clean bases (bp)	%GC
O. cansus	CNS0589733	sch2021166	B166	29,754,171	8,926,251,300	51
O. cansus	CNS0589734	sch2021180	B180	21,688,457	6,506,537,100	50
O. cansus	CNS0589735	sch2021183	B183	24,637,541	7,391,262,300	51
O. cansus	CNS0589736	sch2021184	B184	19,959,147	5,987,744,100	50
O. cansus	CNS0589737	sch2021186	B186	27,408,646	8,222,593,800	50
O. cansus	CNS0589738	sch2021187	B187	20,520,825	6,156,247,500	49
O. cansus	CNS0589739	sch2021189	B189	26,237,870	7,871,361,000	52
O. cansus	CNS0589740	sch2021190	B190	27,549,526	8,264,857,800	51
O. cansus	CNS0589741	sch2021191	B191	21,107,070	6,332,121,000	51
O. cansus	CNS0589742	sch2021237	B237	18,404,541	5,521,362,300	51
O. cansus	CNS0589743	sch2021239	B239	22,790,712	6,837,213,600	51

Species	Field code	Acquisition number	ID	Clean Reads (bp)	Clean bases (bp)	%GC
O. cansus	CNS0589744	sch2021240	B240	21,717,969	6,515,390,700	51
O. cansus	CNS0589745	sch2021245	B245	5,614,055	1,684,216,500	51
O. cansus	CNS0589746	sch2021246	B246	19,864,104	5,959,231,200	50
O. curzoniae	CNS0589747	sch2021660	B660	22,716,181	6,814,854,300	50
O. curzoniae	CNS0589748	sch2021661	B661	25,730,387	7,719,116,100	51
O. curzoniae	CNS0589749	sch2021662	B662	30,078,105	9,023,431,500	51
O. curzoniae	CNS0589750	sch2021663	B663	23,848,151	7,154,445,300	51
O. curzoniae	CNS0589751	sch2021665	B665	21,119,447	6,335,834,100	51
O. curzoniae	CNS0589752	sch2021667	B667	24,387,737	7,316,321,100	52
O. curzoniae	CNS0589753	sch2021682	B682	30,430,509	9,129,152,700	50
O. curzoniae	CNS0589754	sch2021686	B686	26,072,900	7,821,870,000	51
O. curzoniae	CNS0589755	sch2021688	B688	22,085,554	6,625,666,200	51
O. curzoniae	CNS0589756	sch2021689	B689	24,939,599	7,481,879,700	50
O. curzoniae	CNS0589757	sch2021690	B690	25,499,043	7,649,712,900	50
O. curzoniae	CNS0589758	sch2021692	B692	23,444,292	7,033,287,600	50
O. curzoniae	CNS0589759	sch2021694	B694	25,701,282	7,710,384,600	51
O. curzoniae	CNS0589760	sch2021695	B695	22,332,055	6,699,616,500	51

Supplementary Table 3 GO enrichment analysis results of SQ' upregulated genes in SQ vs. WL difference analysis.

GO_ID	Pathway	Enrichment FDR	nGenes	Pathway Genes	Fold Enrichment
GO:0032787	Monocarboxylic acid metabolic process	0.000089	21	207	3.5532
GO:0030163	Protein catabolic process	0.000074	31	393	2.7627
GO:0044265	Cellular macromolecule catabolic process	0.000108	34	469	2.5391
GO:0009057	Macromolecule catabolic process	0.000040	39	546	2.5017
GO:1901565	Organonitrogen compound catabolic process	0.000074	37	520	2.4921
GO:1901575	Organic substance catabolic process	0.000012	53	830	2.2365
GO:0005829	Cytosol	0.000000	100	1577	2.2209
GO:0044281	Small molecule metabolic process	0.000075	46	728	2.2131
GO:0044248	Cellular catabolic process	0.000013	54	859	2.2017
GO:0009056	Catabolic process	0.000004	62	999	2.1737
GO:0033036	Macromolecule localization	0.000052	63	1128	1.9561
GO:0012505	Endomembrane system	0.000003	83	1510	1.9252
GO:0042221	Response to chemical	0.000067	68	1267	1.8797
GO:0005737	Cytoplasm	0.000000	228	4364	1.8299
GO:0036211	Protein modification process	0.000075	88	1821	1.6925
GO:0006464	Cellular protein modification process	0.000075	88	1821	1.6925
GO:0048522	Positive regulation of cellular process	0.000013	103	2137	1.6881
GO:0043231	Intracellular membrane-bounded organelle	0.000000	226	4729	1.6738
GO:0016043	Cellular component organization	0.000010	111	2339	1.6621
GO:0048518	Positive regulation of biological process	0.000010	112	2365	1.6586
GO:0071840	Cellular component organization or biogenesis	0.000012	113	2411	1.6415
GO:0043227	Membrane-bounded organelle	0.000000	229	4922	1.6295
GO:0044267	Cellular protein metabolic process	0.000040	108	2339	1.6172

GO_ID	Pathway	Enrichment FDR	nGenes	Pathway Genes	Fold Enrichment
GO:0019538	Protein metabolic process	0.000012	126	2784	1.5851
GO:1901564	Organonitrogen compound metabolic process	0.000004	145	3260	1.5578
GO:0051179	Localization	0.000047	125	2845	1.5388
GO:0005634	Nucleus	0.000074	129	3000	1.5060
GO:0044260	Cellular macromolecule metabolic process	0.000010	163	3882	1.4706
GO:0006807	Nitrogen compound metabolic process	0.000040	194	4976	1.3655
GO:0043170	Macromolecule metabolic process	0.000075	184	4723	1.3645

Supplementary Table 4 GO enrichment analysis results of REG' upregulated in REG vs. WL difference analysis.

GO_ID	Pathway	Enrichment FDR	nGenes	Pathway Genes	Fold Enrichment
GO:0048662	Negative regulation of smooth muscle cell proliferation	0.00041	6	19	14.82265782
GO:0031346	Positive regulation of cell projection organization	0.00051	11	88	5.867302053
GO:0120035	Regulation of plasma membrane bounded cell projection organization	0.00008	16	157	4.783532884
GO:0031344	Regulation of cell projection organization	0.00008	16	158	4.753257359
GO:0051130	Positive regulation of cellular component organization	0.00051	24	384	2.933651026
GO:0019752	Carboxylic acid metabolic process	0.00118	23	378	2.856041211
GO:0048699	Generation of neurons	0.00264	21	346	2.848863424
GO:0043436	Oxoacid metabolic process	0.00135	23	383	2.818756078
GO:0006082	Organic acid metabolic process	0.00189	23	392	2.754039739
GO:0022008	Neurogenesis	0.00349	22	382	2.703259585
GO:0007399	Nervous system development	0.00012	32	562	2.672650045
GO:0048468	Cell development	0.00131	28	523	2.512955373
GO:0051128	Regulation of cellular component organization	0.00005	43	845	2.388582137

GO_ID	Pathway	Enrichment FDR	nGenes	Pathway Genes	Fold Enrichment
GO:0042802	Identical protein binding	0.00022	44	948	2.17857629
GO:0048869	Cellular developmental process	0.00009	51	1136	2.107270456
GO:0030154	Cell differentiation	0.00016	49	1097	2.096611126
GO:0005829	Cytosol	0.00000	70	1577	2.083506119
GO:0071702	Organic substance transport	0.00293	38	868	2.054907631
GO:0007275	Multicellular organism development	0.00006	63	1516	1.950607015
GO:0048856	Anatomical structure development	0.00002	72	1743	1.938936307
GO:0032502	Developmental process	0.00001	77	1879	1.923500833
GO:0048731	System development	0.00030	57	1398	1.913798094
GO:0016043	Cellular component organization	0.00002	89	2339	1.786027816
GO:0032501	Multicellular organismal process	0.00005	82	2170	1.773709745
GO:0071840	Cellular component organization or biogenesis	0.00002	90	2411	1.752159883
GO:0006996	Organelle organization	0.00349	56	1510	1.740762463
GO:0005737	Cytoplasm	0.00000	160	4364	1.720931858
GO:0048518	Positive regulation of biological process	0.00063	82	2365	1.627463064
GO:0043231	Intracellular membrane-bounded organelle	0.00005	146	4729	1.449145443
GO:0043227	Membrane-bounded organelle	0.00005	150	4922	1.43046779

Supplementary Table 5 GO enrichment analysis results of SQ' upregulated in SQ vs. REG difference analysis.

GO_ID	Pathway	Enrichment FDR	nGenes	Pathway Genes	Fold Enrichment
GO:0047429	Nucleoside-triphosphate diphosphatase activity	0.01677	2	8	151.5555556
GO:0022625	Cytosolic large ribosomal subunit	0.01822	2	15	80.82962963
GO:0022626	Cytosolic ribosome	0.01822	2	15	80.82962963
GO:0015934	Large ribosomal subunit	0.01486	3	41	44.35772358

GO_ID	Pathway	Enrichment FDR	nGenes	Pathway Genes	Fold Enrichment
GO:0097190	Apoptotic signaling pathway	0.04771	2	30	40.41481481
GO:0044391	Ribosomal subunit	0.01822	3	80	22.73333333
GO:0005829	Cytosol	0.03409	3	107	16.99688474
GO:0019439	Aromatic compound catabolic process	0.01822	4	187	12.96732026
GO:0044270	Cellular nitrogen compound catabolic process	0.01822	4	187	12.96732026
GO:0046700	Heterocycle catabolic process	0.01822	4	187	12.96732026
GO:1901361	Organic cyclic compound catabolic process	0.01822	4	194	12.49942726
GO:0006974	Cellular response to DNA damage stimulus	0.01486	6	381	9.546806649
GO:0006281	DNA repair	0.01822	5	333	9.102435769
GO:0034660	NcRNA metabolic process	0.04850	4	280	8.66031746
GO:0033554	Cellular response to stress	0.01822	6	486	7.484224966
GO:0006396	RNA processing	0.02678	6	606	6.00220022
GO:0006950	Response to stress	0.03182	7	883	4.805838681

Supplementary Table 6 GO enrichment analysis results of the same upregulated genes in SQ vs. WL and REG vs. WL.

GO_ID	Pathway	Enrichment FDR	nGenes	Pathway Genes	Fold Enrichment
GO:0034235	GPI anchor binding	0.01629	2	2	81.24873096
GO:0097750	Endosome membrane tubulation	0.01629	2	2	81.24873096
GO:0140285	Endosome fission	0.01629	2	2	81.24873096
GO:0010976	Positive regulation of neuron projection development	0.00579	5	30	13.54145516
GO:0031346	Positive regulation of cell projection organization	0.00366	8	88	7.386248269
GO:0031252	Cell leading edge	0.01629	8	125	5.199918782
GO:0120035	Regulation of plasma membrane bounded cell projection	0.00543	10	157	5.175078405

GO_ID	Pathway	Enrichment FDR	nGenes	Pathway Genes	Fold Enrichment
	organization				
GO:0031344	Regulation of cell projection organization	0.00543	10	158	5.142324745
GO:0006974	Cellular response to DNA damage stimulus	0.01521	15	391	3.116958988
GO:0007399	Nervous system development	0.00272	21	562	3.035984609
GO:0033554	Cellular response to stress	0.00270	26	759	2.783223986
GO:0051128	Regulation of cellular component organization	0.01629	24	845	2.307656264
GO:0030554	Adenyl nucleotide binding	0.00915	28	1022	2.225992629
GO:0005524	ATP binding	0.01335	27	992	2.211406992
GO:0032559	Adenyl ribonucleotide binding	0.01629	27	1016	2.159169032
GO:0005829	Cytosol	0.00270	41	1577	2.112363963
GO:0016740	Transferase activity	0.00531	38	1532	2.015307948
GO:0140096	Catalytic activity, acting on a protein	0.01629	36	1545	1.893174314
GO:0036211	Protein modification process	0.00695	42	1821	1.873941077
GO:0006464	Cellular protein modification process	0.00695	42	1821	1.873941077
GO:0016043	Cellular component organization	0.00270	53	2339	1.841035802
GO:0071840	Cellular component organization or biogenesis	0.00270	54	2411	1.819755899
GO:0048856	Anatomical structure development	0.01629	39	1743	1.817957836
GO:0005737	Cytoplasm	0.00000	96	4364	1.787323138
GO:0043412	Macromolecule modification	0.01629	42	1919	1.778242158
GO:0044267	Cellular protein metabolic process	0.00531	51	2339	1.771562753
GO:0019538	Protein metabolic process	0.00322	59	2784	1.721866066
GO:1901564	Organonitrogen compound metabolic process	0.00270	67	3260	1.669835882
GO:0043231	Intracellular membrane-bounded organelle	0.00322	87	4729	1.494742989
GO:0043227	Membrane-bounded organelle	0.00366	89	4922	1.46914609

Supplementary Table 7 GO enrichment analysis results of SQ' downregulated genes in SQ vs. WL difference analysis.

GO_ID	Pathway	Enrichment FDR	nGenes	Pathway Genes	Fold Enrichment
GO:1902531	Regulation of intracellular signal transduction	0.00004	47	643	2.249916258
GO:0035556	Intracellular signal transduction	0.00000	75	1123	2.05570587
GO:0009966	Regulation of signal transduction	0.00001	71	1127	1.939161149
GO:0005886	Plasma membrane	0.00000	105	1708	1.892260404
GO:0010646	Regulation of cell communication	0.00001	75	1220	1.892260404
GO:0023051	Regulation of signaling	0.00002	75	1227	1.881465112
GO:0071944	Cell periphery	0.00000	110	1855	1.825274725
GO:0048583	Regulation of response to stimulus	0.00001	86	1470	1.800779696
GO:0048522	Positive regulation of cellular process	0.00000	119	2137	1.714043771
GO:0005654	Nucleoplasm	0.00003	102	1890	1.661184371
GO:0036211	Protein modification process	0.00005	98	1821	1.656515862
GO:0006464	Cellular protein modification process	0.00005	98	1821	1.656515862
GO:0031981	Nuclear lumen	0.00001	111	2075	1.646585728
GO:0048518	Positive regulation of biological process	0.00000	126	2365	1.639905676
GO:0007154	Cell communication	0.00000	147	2801	1.615413451
GO:0023052	Signaling	0.00000	144	2769	1.600733394
GO:0007165	Signal transduction	0.00000	135	2609	1.59271899
GO:0031974	Membrane-enclosed lumen	0.00005	114	2226	1.576373626
GO:0043233	Organelle lumen	0.00005	114	2226	1.576373626
GO:0070013	Intracellular organelle lumen	0.00005	114	2226	1.576373626
GO:0005634	Nucleus	0.00000	153	3000	1.569819231
GO:0044267	Cellular protein metabolic process	0.00005	119	2339	1.566016049
GO:0005737	Cytoplasm	0.00000	219	4364	1.544681132

GO_ID	Pathway	Enrichment FDR	nGenes	Pathway Genes	Fold Enrichment
GO:0051716	Cellular response to stimulus	0.00001	156	3214	1.494026136
GO:0019538	Protein metabolic process	0.00007	135	2784	1.492601956
GO:0019222	Regulation of metabolic process	0.00012	132	2736	1.485037112
GO:0043227	Membrane-bounded organelle	0.00000	237	4922	1.482129685
GO:0043231	Intracellular membrane-bounded organelle	0.00000	227	4729	1.477528995
GO:0050896	Response to stimulus	0.00001	176	3764	1.439270825
GO:0016020	Membrane	0.00005	172	3760	1.408056465

Supplementary Table 8 GO enrichment analysis results of REG' downregulated genes in REG vs. WL difference analysis.

GO_ID	Pathway	Enrichment FDR	nGenes	Pathway Genes	Fold Enrichment
GO:0060759	Regulation of response to cytokine stimulus	0.00598	9	59	5.587169841
GO:0071345	Cellular response to cytokine stimulus	0.00863	18	227	2.904343794
GO:0098662	Inorganic cation transmembrane transport	0.00825	20	267	2.743595677
GO:0098660	Inorganic ion transmembrane transport	0.00825	21	289	2.661477675
GO:0008092	Cytoskeletal protein binding	0.00505	31	501	2.266341459
GO:0006812	Cation transport	0.00809	31	521	2.179341787
GO:0007010	Cytoskeleton organization	0.00520	36	628	2.099637074
GO:0035556	Intracellular signal transduction	0.00033	59	1123	1.924303771
GO:0010646	Regulation of cell communication	0.00093	61	1220	1.831350114
GO:0032879	Regulation of localization	0.00505	51	1020	1.831350114
GO:0023051	Regulation of signaling	0.00104	61	1227	1.820902314
GO:0005829	Cytosol	0.00027	76	1577	1.765156737
GO:0051234	Establishment of localization	0.00000	103	2151	1.753873192
GO:0071944	Cell periphery	0.00008	88	1855	1.737561295

GO_ID	Pathway	Enrichment FDR	nGenes	Pathway Genes	Fold Enrichment
GO:0006810	Transport	0.00002	98	2074	1.730687668
GO:0005886	Plasma membrane	0.00033	80	1708	1.715550458
GO:0016740	Transferase activity	0.00505	69	1532	1.649649581
GO:0051179	Localization	0.00002	124	2845	1.596396585
GO:0048519	Negative regulation of biological process	0.00325	86	2006	1.570250347
GO:0007154	Cell communication	0.00011	119	2801	1.55609185
GO:0048523	Negative regulation of cellular process	0.00961	77	1826	1.544512145
GO:0005737	Cytoplasm	0.00000	182	4364	1.527523927
GO:0005634	Nucleus	0.00022	124	3000	1.513916095
GO:0023052	Signaling	0.00068	114	2769	1.507937256
GO:0032501	Multicellular organismal process	0.00809	89	2170	1.502213458
GO:0043227	Membrane-bounded organelle	0.00000	199	4922	1.480856045
GO:0048518	Positive regulation of biological process	0.00829	95	2365	1.471274933
GO:0007165	Signal transduction	0.00573	104	2609	1.460026155
GO:0043231	Intracellular membrane-bounded organelle	0.00000	188	4729	1.456095671
GO:0016020	Membrane	0.00865	138	3760	1.344288914

Supplementary Table 9 GO enrichment analysis results of REG' downregulated genes in SQ vs. REG difference analysis.

GO_ID	Pathway	Enrichment FDR	nGenes	Pathway Genes	Fold Enrichment
GO:0030140	Trans-Golgi network transport vesicle	0.04673	2	12	55.57638889
GO:0007030	Golgi organization	0.04673	3	51	19.61519608
GO:0007346	Regulation of mitotic cell cycle	0.02551	5	177	9.41972693
GO:0000278	Mitotic cell cycle	0.00519	8	352	7.578598485
GO:0051726	Regulation of cell cycle	0.00367	9	398	7.540515075

GO_ID	Pathway	Enrichment FDR	nGenes	Pathway Genes	Fold Enrichment
GO:0000226	Microtubule cytoskeleton organization	0.02551	6	282	7.094858156
GO:0099512	Supramolecular fiber	0.02551	6	282	7.094858156
GO:0099081	Supramolecular polymer	0.02551	6	285	7.020175439
GO:0099080	Supramolecular complex	0.01503	8	432	6.175154321
GO:0007049	Cell cycle	0.00519	10	606	5.502612761
GO:0007017	Microtubule-based process	0.03307	7	436	5.353688838
GO:0007010	Cytoskeleton organization	0.04673	8	628	4.247876858
GO:0031974	Membrane-enclosed lumen	0.02551	17	2226	2.546626984
GO:0043233	Organelle lumen	0.02551	17	2226	2.546626984
GO:0070013	Intracellular organelle lumen	0.02551	17	2226	2.546626984
GO:0043228	Non-membrane-bounded organelle	0.04673	15	2017	2.479858701
GO:0043232	Intracellular non-membrane-bounded organelle	0.04673	15	2017	2.479858701
GO:0005634	Nucleus	0.04831	19	3000	2.111902778
GO:0043231	Intracellular membrane-bounded organelle	0.02551	27	4729	1.903864453
GO:0043227	Membrane-bounded organelle	0.02551	27	4922	1.829210687

Supplementary Table 10 GO enrichment analysis results of the same downregulated genes in SQ vs. WL and REG vs. WL.

GO_ID	Pathway	Enrichment FDR	nGenes	Pathway Genes	Fold Enrichment
GO:0010961	Cellular magnesium ion homeostasis	0.03615	2	2	74.44651163
GO:0043394	Proteoglycan binding	0.03615	3	9	24.81550388
GO:0048015	Phosphatidylinositol-mediated signaling	0.03605	6	60	7.444651163
GO:0048017	Inositol lipid-mediated signaling	0.03615	6	63	7.090143965
GO:0035556	Intracellular signal transduction	0.01664	33	1123	2.187653503
GO:0071944	Cell periphery	0.01664	46	1855	1.846112957

GO_ID	Pathway	Enrichment FDR	nGenes	Pathway Genes	Fold Enrichment
GO:0005886	Plasma membrane	0.03605	41	1708	1.787064975
GO:0051234	Establishment of localization	0.02442	50	2151	1.730509336
GO:0006810	Transport	0.02548	48	2074	1.722966518
GO:0007154	Cell communication	0.01664	63	2801	1.674448494
GO:0023052	Signaling	0.01704	61	2769	1.640027883
GO:0007165	Signal transduction	0.02548	57	2609	1.626466525
GO:0051179	Localization	0.02548	61	2845	1.596216945
GO:0005737	Cytoplasm	0.00173	92	4364	1.569449833
GO:0016020	Membrane	0.01664	77	3760	1.52456952
GO:0043227	Membrane-bounded organelle	0.02442	93	4922	1.406648838

Supplementary Table 11 GO function enrichment information for hub genes.

GO_ID	Pathway	Enrichment FDR	nGenes	Pathway Genes	Fold Enrichment
GO:0005046	KDEL sequence binding	0.03077	2	2	59.06273063
GO:0043931	Ossification involved in bone maturation	0.02587	3	7	25.31259884
GO:0048799	Animal organ maturation	0.02929	3	8	22.14852399
GO:0070977	Bone maturation	0.02929	3	8	22.14852399
GO:0048662	Negative regulation of smooth muscle cell proliferation	0.02929	4	19	12.43425908
GO:0010008	Endosome membrane	0.00339	11	117	5.552906298
GO:0030659	Cytoplasmic vesicle membrane	0.00360	14	195	4.240401173
GO:0007507	Heart development	0.03077	9	126	4.218766473
GO:0012506	Vesicle membrane	0.00413	14	200	4.134391144
GO:0005543	Phospholipid binding	0.01862	13	215	3.571234875
GO:1901137	Carbohydrate derivative biosynthetic process	0.02929	14	271	3.051211176

GO_ID	Pathway	Enrichment FDR	nGenes	Pathway Genes	Fold Enrichment
GO:0005768	Endosome	0.02072	16	317	2.981084196
GO:0008289	Lipid binding	0.02929	16	340	2.779422618
GO:0031984	Organelle subcompartment	0.02587	17	362	2.773664145
GO:0098588	Bounding membrane of organelle	0.01862	19	412	2.723766704
GO:0046907	Intracellular transport	0.01167	25	592	2.494203151
GO:1901135	Carbohydrate derivative metabolic process	0.04326	18	437	2.43278982
GO:0031410	Cytoplasmic vesicle	0.02929	22	565	2.299787741
GO:0097708	Intracellular vesicle	0.02929	22	566	2.295724512
GO:0051649	Establishment of localization in cell	0.01653	29	766	2.236056381
GO:0016192	Vesicle-mediated transport	0.04238	22	590	2.202339108
GO:0051641	Cellular localization	0.00273	41	1133	2.137309758
GO:0031090	Organelle membrane	0.01862	31	877	2.087736202
GO:0008104	Protein localization	0.01862	32	912	2.072376513
GO:0009966	Regulation of signal transduction	0.01862	37	1127	1.939060367
GO:0033036	Macromolecule localization	0.01862	37	1128	1.937341341
GO:0023051	Regulation of signaling	0.01653	40	1227	1.925435391
GO:0010646	Regulation of cell communication	0.01862	39	1220	1.888070897
GO:0005737	Cytoplasm	0.00000	131	4364	1.772964645
GO:0048583	Regulation of response to stimulus	0.02569	44	1470	1.767864046
GO:0012505	Endomembrane system	0.02929	44	1510	1.72103321
GO:0005829	Cytosol	0.03279	45	1577	1.685366441
GO:0006810	Transport	0.02929	56	2074	1.594750682
GO:0043231	Intracellular membrane-bounded organelle	0.00000	127	4729	1.586163415
GO:0051234	Establishment of localization	0.03279	57	2151	1.565121174
GO:0043227	Membrane-bounded organelle	0.00000	130	4922	1.559966473

GO_ID	Pathway	Enrichment FDR	nGenes	Pathway Genes	Fold Enrichment
GO:0071840	Cellular component organization or biogenesis	0.03640	62	2411	1.518825922
GO:0032991	Protein-containing complex	0.03279	64	2499	1.512610948
GO:0051179	Localization	0.02839	72	2845	1.494733429
GO:0016020	Membrane	0.02587	90	3760	1.413735574

Supplementary Table 12 Number of modules and genes divided before merging

Module color	Gene numbers	Module color	Gene numbers
Black	254	Pink	190
Blue	1250	Purple	165
Brown	1207	Red	316
Cyan	56	Salmon	93
Green	381	Tan	129
Green yellow	131	Turquoise	1699
Grey	10	Yellow	474
Magenta	185		

Supplementary Table 13 The number of genes in the merged module.

Module color	Gene numbers	Module color	Gene numbers
Black	439	Pink	450
Blue	2457	Purple	165
Cyan	56	Red	2015
Green	855	Salmon	93
Grey	10		

Supplementary Table 14 Pearson's correlation coefficients between module expression and phenotypic traits.

Character / Module	Body weight	Bogy length	Ear length	Metaleg length	Elevation height	Gender type	Species type
MEblue	-0.74<br>(6e-06)	-0.74<br>(8e-06)	-0.33<br>(0.09)	0.081<br>(0.7)	-0.8<br>(3e-07)	0.14<br>(0.5)	-0.84<br>(2e-08)
MEgreen	-0.31<br>(0.1)	-0.36<br>(0.06)	-0.083<br>(0.7)	-0.06<br>(0.8)	-0.58<br>(0.001)	0.11<br>(0.6)	-0.54<br>(0.003)
MEpink	0.32<br>(0.1)	0.33<br>(0.09)	0.23<br>(0.2)	0.15<br>(0.4)	0.21<br>(0.3)	-0.19<br>(0.3)	0.25<br>(0.3)
MEred	0.88<br>(9e-10)	0.87<br>(2e-09)	0.43<br>(0.02)	-0.19<br>(0.3)	0.8<br>(4e-07)	0.11<br>(0.6)	0.89<br>(2e-10)
MEpurple	0.13<br>(0.5)	-0.03<br>(0.9)	0.099<br>(0.6)	0.0015<br>(1)	0.091<br>(0.6)	-0.15<br>(0.4)	0.055<br>(0.8)
MEsalmon	0.093<br>(0.6)	0.092<br>(0.6)	0.019<br>(0.9)	0.18<br>(0.4)	0.21<br>(0.3)	-0.25<br>(0.2)	0.18<br>(0.4)
MEblack	-0.36<br>(0.06)	-0.29<br>(0.1)	-0.2<br>(0.3)	0.26<br>(0.2)	-0.056<br>(0.8)	-0.21<br>(0.3)	-0.15<br>(0.4)
MEcyan	-0.13<br>(0.5)	-0.3<br>(0.1)	-0.45<br>(0.02)	-0.29<br>(0.1)	-0.08<br>(0.7)	-0.59<br>(0.001)	-0.17<br>(0.4)
MEgrey	0.029<br>(0.9)	0.046<br>(0.8)	0.063<br>(0.8)	0.13<br>(0.5)	-0.005<br>(0.8)	-0.36<br>(0.06)	-0.053<br>(0.8)

Note that the corresponding P value of Pearson's correlation coefficient is shown in the bottom parenthesis.
